# Intermittent furosemide administration in patients with or at risk for acute kidney injury: Meta-analysis of randomized trials

**DOI:** 10.1371/journal.pone.0196088

**Published:** 2018-04-24

**Authors:** Tiziana Bove, Alessandro Belletti, Alessandro Putzu, Simone Pappacena, Giuseppe Denaro, Giovanni Landoni, Sean M. Bagshaw, Alberto Zangrillo

**Affiliations:** 1 Department of Anesthesia and Intensive Care, IRCCS San Raffaele Scientific Institute, Milan, Italy; 2 Department of Cardiovascular Anesthesia and Intensive Care, Fondazione Cardiocentro Ticino, Lugano, Switzerland; 3 Vita-Salute San Raffaele University, Milan, Italy; 4 Department of Critical Care Medicine, Faculty of Medicine and Dentistry, University of Alberta, Edmonton, Canada; University of Sao Paulo Medical School, BRAZIL

## Abstract

**Background:**

Furosemide is the most common loop diuretic used worldwide. The off-label administration of furosemide bolus(es) for the prevention or to reverse acute kidney injury (AKI) is widespread but not supported by available evidence. We conducted a meta-analysis of randomized trials (RCTs) to investigate whether bolus furosemide to prevent or treat AKI is detrimental on patients’ survival.

**Methods:**

Electronic databases were searched through October 2017 for RCTs comparing bolus furosemide administration versus any comparator in patients with or at risk for AKI. The primary endpoint was all-cause longest follow-up mortality. Secondary endpoints included new or worsening AKI, receipt of renal replacement therapy, length of hospital stay, and peak serum creatinine after randomization.

**Results:**

A total of 28 studies randomizing 3,228 patients were included in the analysis. We found no difference in mortality between the two groups (143/892 [16%] in the furosemide group versus 141/881 [16%] in the control group; odds ratio [OR], 0.84; 95% confidence interval [CI], 0.63 to 1.13; p = 0.25). No significant differences in secondary outcomes were found. A significant improvement in survival was found in the subgroup of patients receiving furosemide bolus(es) as a preventive measure (43/613 [7.0%] versus 67/619 [10.8%], OR 0.62; 95% CI, 0.41 to 0.94; p = 0.03)

**Conclusions:**

Intermittent furosemide administration is not associated with an increased mortality in patients with or at risk for AKI, although it may reduce mortality when used as a preventive measure. Future high-quality RCTs are needed to define the role of loop diuretics in AKI prevention and management.

**Trial registration:**

The study protocol was registered on PROSPERO database for systematic reviews (Registration no. CRD42017078607 – http://www.crd.york.ac.uk/PROSPERO/display_record.php?ID=CRD42017078607).

## Introduction

Acute kidney injury (AKI) is a common complication in hospitalized patients, with an overall estimated incidence of around 10%, increasing up to 60% among critically ill patients admitted to an intensive care unit (ICU) [1]. Of these, 6% develop severe AKI, with approximately 70% receiving acute renal replacement therapy (RRT)[2]. A relationship between renal failure and increased short- and long-term morbidity and mortality is well recognized across several clinical settings [1,3].

Currently, there are few interventions or medications that can alter the clinical course of AKI and favorably modify the outcome of critically ill patients once AKI occurred [4,5].

The use of loop diuretics in critically ill patients with AKI is a long standing and widespread clinical practice [6]. The rationale for the use of loop diuretics includes many aspects of their action, including an increase of tubular flow, a reduction in oxygen consumption and ischemic injury, and a reduction of TNF-induced apoptosis [7,8,9].

Furosemide remains the most common loop diuretic prescribed in critically ill patients [6,10,11]. Experimental studies have suggested that the infusion of low doses of furosemide can reduce the apoptosis phenomena induced by ischemia/reperfusion and gene transcription associated therewith [12,13].

On one hand, some small studies have suggested that diuretics can reduce the severity of acute renal failure transforming it from oliguric to not oliguric, reducing the duration of AKI, improving the speed of recovery of renal function and probably reducing the need of renal replacement treatment [14,15,16,17]. Furthermore, furosemide may also be helpful in the management of volume overload and electrolyte homeostasis, which could be ultimately related to AKI outcome [18,19].

On the other hand, several observational studies suggested that the use of diuretics in critically ill patients with AKI might not be associated with improvement in clinically relevant outcomes, and may even increase mortality [10,11]. A possible detrimental effect of loops diuretics administration has been suggested also for heart failure patients [20]. In addition, some in vitro studies, where blood mononuclear cells were stimulated with lipopolysaccharide, have revealed that high concentrations of furosemide could have cytotoxic and immunosuppressive effects characterized by reduced expression of interleukin-6, interleukin-8, and tumor necrosis factor-alpha [21].

In clinical practice, it is common experience that intermittent (bolus) furosemide administration is frequently the first strategy applied by clinicians when facing patients with early AKI, especially when oligo-anuria is present. However, such strategy is not currently supported by evidence-based medicine, and, in some settings, has been associated with harm [22,23].

Thus, we decided to carry out a systematic review and meta-analysis of all randomized clinical trials ever performed on furosemide bolus versus any comparator in any clinical AKI setting to evaluate its effect on survival and on clinically relevant outcomes.

## Methods

This study is a systematic review and meta-analysis conducted in keeping with Preferred Reporting Items for Systematic Reviews and Meta-Analyses (PRISMA) guidelines [24,25,26]. The study protocol was registered on PROSPERO database for systematic reviews (Registration no. CRD42017078607) [27]. The PRISMA Checklist is available as S1 Checklist.

This study was supported by a grant from the Italian Medicines Agency (AIFA–Grant no. FARM12JFX9). The funders had no role in study design, data collection and analysis, decision to publish, or preparation of the manuscript.

### Search strategy

Pertinent studies were searched independently in BioMedCentral, PubMed, EMBASE, and the Cochrane Central Register of clinical trials by four trained investigators (search last updated October 15th, 2017). The PubMed search strategy used to include any randomized study ever performed with furosemide in patients with or at risk for AKI [28] is available in S1 Appendix. In addition, we employed backward snowballing (i.e., scanning of references of retrieved articles and pertinent reviews) and contacted the international experts and the manufacturers for further studies. No language restriction was enforced.

### Study selection

References, obtained from database and literature searches, were examined first at a title/abstract level independently by four investigators, with divergences resolved by consensus, and then, if potentially pertinent, were retrieved as complete articles.

The following inclusion criteria were used for potentially relevant studies: (1) random allocation to treatment, (2) comparison between furosemide bolus versus any comparator, and (3) critically ill patients. There was no restriction on dose or time of administration. The exclusion criteria were as follows: (1) non-adult studies, (2) studies with a non-parallel design (e.g., crossover) randomized trials, (3) duplicate publications either acknowledged or not (in this case we referred to the first article published while we retrieved data from the article with the longest follow-up available, (4) non-human experimental studies, (5) studies with no data on outcome of interests; and (6) oral furosemide administration.

Two investigators independently assessed the compliance to selection criteria and selected the studies for the final analysis, with divergences finally resolved by consensus.

### Data abstraction and study endpoint

Baseline characteristics, procedural, and outcome data were abstracted independently by four trained investigators; the divergences were resolved by consensus. Specifically, we extracted potential sources of significant clinical heterogeneity, such as study design, sample size, clinical setting/indication, furosemide bolus dose, control treatment, and follow-up duration, as well as primary study endpoint and other key outcomes. Corresponding author of original authors were contacted in cases of missing data on outcome of interests.

### Risk of bias assessment

The internal validity and risk of bias of included trials was appraised by two independent reviewers according to the Risk of Bias Assessment Tool developed by the Cochrane collaboration [29, 30] that assesses the adequacy of randomization sequence generation, the concealment of treatment allocation, blinding of participating subjects, treating personnel and outcome assessors, complete reporting of outcome, possible selective outcome reporting, possible other sources of bias, and provide a final judgement on the overall risk of bias. Publication bias were assessed by visually inspecting funnel plots for pooled analyses containing >10 studies [31].

### Data analysis and synthesis

Computations were performed with RevMan (Review Manager, Version 5.3. Copenhagen: The Nordic Cochrane Centre, The Cochrane Collaboration, 2014; available at http://community.cochrane.org/tools/review-production-tools/revman-5/revman-5-download). Hypothesis of statistical heterogeneity was tested by means of Cochran Q test, with statistical significance set at the two-tailed 0.10 level, whereas extent of statistical consistency was measured with I^2^, defined as 100% X (Q-df)/Q, where Q is Cochran’s heterogeneity statistic and the degrees of freedom (df). Binary outcomes from individual studies were analyzed to compute individual and pooled odds ratio (OR) with pertinent 95% confidence intervals (CIs), fitting a fixed-effect model in case of low statistical inconsistency (I^2^ <25%) or with random-effect model (which better accommodates clinical and statistical variations) in case of moderate or high statistical inconsistency (I^2^ ≥25%). Weighted mean difference (WMD) and 95% CIs were computed for continuous variables using the same methods as just described. For individual studies reporting continuous outcomes as median and range or median and interquartile range, mean and standard deviation were estimated using equations elaborated by Wan and colleagues [32].

The primary endpoint was all-cause longest follow-up mortality. The pre-specified secondary endpoint were 28/30-days mortality, new/worsening AKI, receipt of RRT, length of hospital stay, and peak serum creatinine after randomization. Outcomes were defined as per-original author’s definition.

Sensitivity analyses were performed by sequentially removing each study and reanalyzing the remaining dataset (producing a new analysis for each study removed), by selecting an individual subset (defined by setting, control drug, and indication for treatment [e.g., prevention versus treatment of AKI]), and by analyzing only data from studies with low risk of bias.

Statistical significance was set at the two-tailed 0.05 level for hypothesis testing. Unadjusted p values are reported throughout.

We performed pre-defined trial sequential analysis (TSA) [33,34], with the intent of maintaining an overall 5% risk of type I error and a 10% risk of type II error, at a power of 90%. We assumed a relative risk reduction of 15% for each outcome. The analysis was conducted using the control event proportion derived from the present meta-analyses. The resulting required information size was further diversity (D2)-adjusted; in case of D^2^ = 0, we performed a sensitivity analysis assuming a D^2^ = 25%. Fixed-effect model was employed. We used the TSA software (TSA Viewer [Computer program], version 0.9.5.5 Beta, Copenhagen Trial Unit, Centre for Clinical Intervention Research, Rigshospitalet, 2016.).

## Results

**Fig 1:**
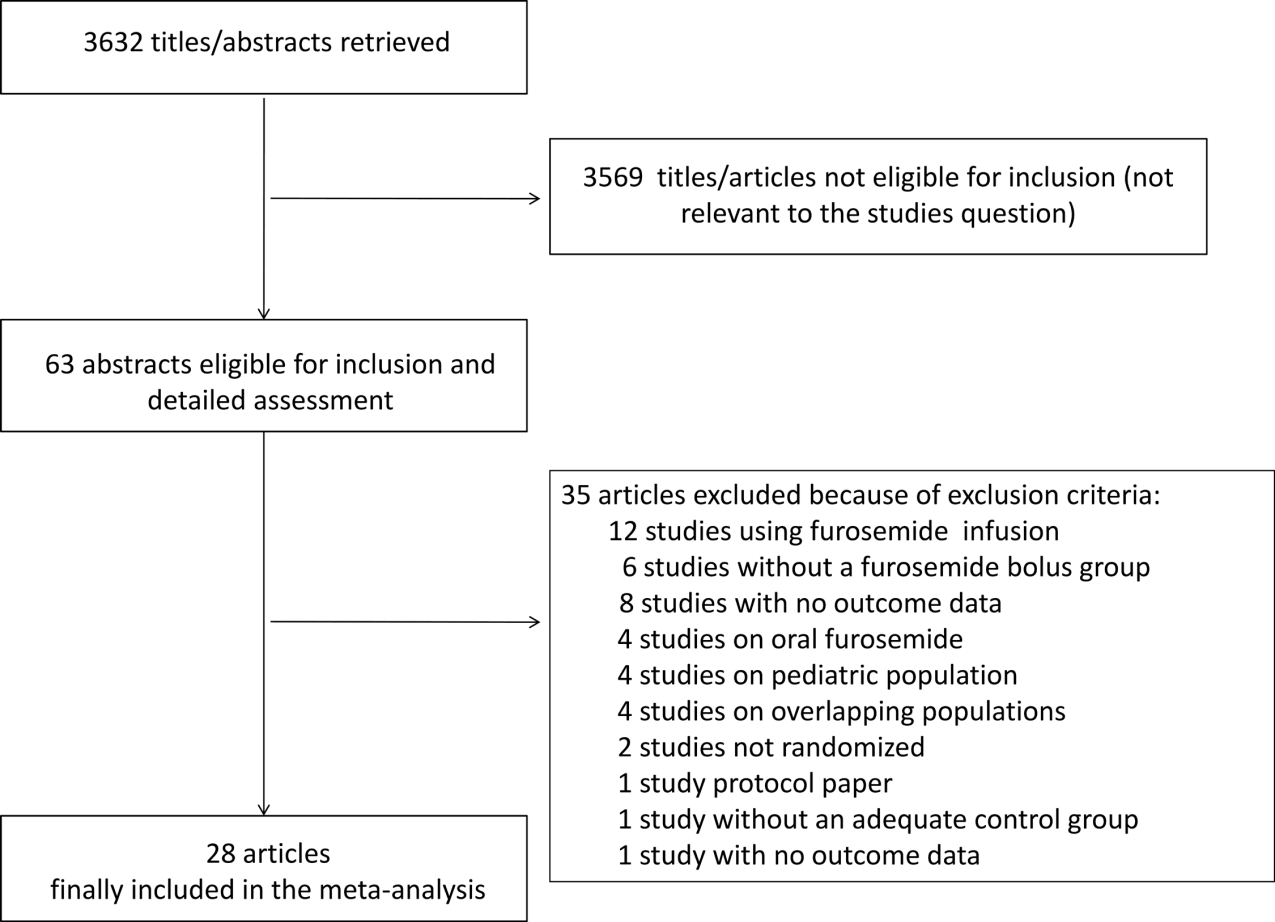
Examined studies.

A total of 3632 references were examined. After exclusions of non-pertinent studies, a total of 63 studies were retrieved as complete articles. Of these, 35 were further excluded, as they did not met inclusion criteria. Details of excluded studies together with reason for exclusion are available in the Table A in S1 Appendix. Finally, a total of 28 studies randomizing 3228 patients were included in the analysis (Fig 1) [15–17,22,35–57].

### Trials’ characteristics

Characteristics of included trials are described in Table 1. Included trials were performed in the settings of AKI either in ICU or general ward, cardiac surgery, acutely decompensated heart failure, and contrast-induced AKI.

**Table 1:** Study characteristics.

First author	Year	Setting	Multi center	Control treatment	N treatment group	N control group	Prevention or treatment?
Ad N [35]	2002	Cardiac surgery	No	Infusion furosemide	39	36	Prevention
Allen LA [36]	2010	Acute decompensated heart failure	No	Infusion furosemide	21	20	Prevention
Bayat F [37]	2015	Cardiac surgery	No	Standard care	42	42	Prevention
Barbanti M [38]	2015	Contrast-Induced AKI	No	Standard care	56	56	Prevention
Briguori C [39]	2011	Contrast-Induced AKI	Yes	Standard care	146	146	Prevention
Brown CB [40]	1981	AKI/ICU	No	Infusion furosemide	28	28	Treatment
Cantarovich F [15]	1973	AKI/ICU	No	Standard care	39	19	Treatment
Copeland JG [41]	1983	Cardiac surgery	No	Infusion furosemide	9	9	Prevention
Dussol B [42]	2006	Contrast-Induced AKI	No	0.9% Saline	79	77	Prevention
Felker BM [43]	2011	Acute decompensated heart failure	No	Infusion furosemide	156	152	Prevention
Gu CQ [44]	2013	Contrast-induced AKI	Yes	Standard care	422	437	Prevention
Karayannopoulos S [16]	1974	AKI/ICU	No	Standard care	10	10	Treatment
Kleinknecht D [17]	1976	AKI/ICU	No	Placebo	33	33	Treatment
Kunt AT [45]	2009	Cardiac surgery	No	Infusion furosemide	50	50	Treatment
Llorens P [46]	2014	Acute decompensated heart failure	No	Infusion furosemide	73	36	Prevention
Marenzi G [47]	2012	Contrast-Induced AKI	Yes	Standard care	87	83	Prevention
Mojtahedzadeh M [48]	2004	AKI/ICU	No	Infusion furosemide	11	11	Prevention
Ostermann M [49]	2007	AKI/ICU	No	Infusion furosemide	26	30	Prevention
Palazzuoli A [50]	2014	Acute decompensated heart failure	No	Infusion furosemide	39	43	Prevention
Schuller D [51]	1997	AKI/ICU	No	Infusion furosemide	19	14	Treatment
Shah RA [52]	2014	Acute decompensated heart failure	No	Infusion furosemide	30	60	Treatment
Shilliday IR [53]	1997	AKI/ICU	No	Placebo	32	30	Treatment
Solomon R [22]	1994	Contrast-Induced AKI	No	Saline (± mannitol)	25	53	Prevention
Thomson MR [54]	2010	Acute decompensated heart failure	No	Infusion furosemide	30	26	Treatment
Usmiani T [55]	2016	Contrast-Induced AKI	No	Standard care	57	63	Prevention
Vargas Hein O [56]	2005	Cardiac surgery	No	Torsemide	14	15	Treatment
Yayla Ç [57]	2015	Acute decompensated heart failure	No	Infusion furosemide	14	15	Prevention

In 14 trials control treatment was represented by continuous furosemide infusion, in 12 trials by placebo/standard treatment, and in 2 further trials by an active pharmacological comparator. Fifteen trials administered furosemide bolus as a preventive measure in patients who had not yet developed AKI, while 11 as treatment of established AKI.

Mortality data were available for 19 trials, with four trials reporting 28/30 days mortality. Data on receipt of RRT were available from 14 trials, data on new onset/worsening AKI from 16 trials, length of hospital stay in 7 trials and peak serum creatinine following randomization in 6 trials.

**Fig 2:**
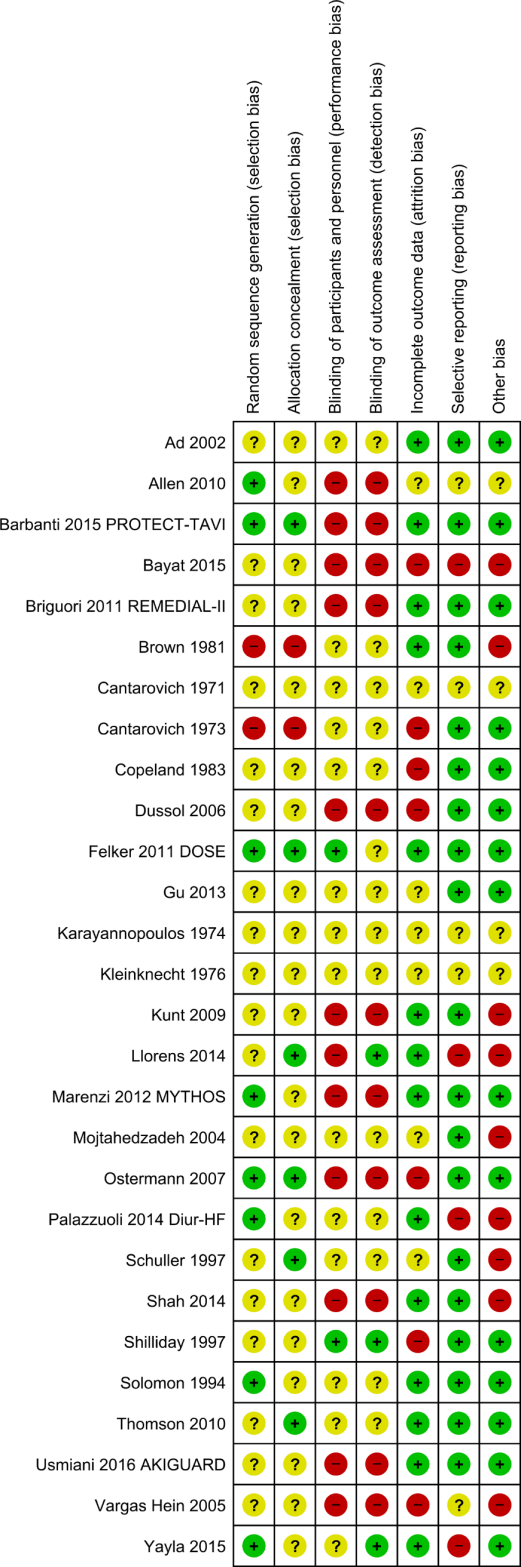
Studies bias.

Overall, risk of bias analysis showed that none of included trials was at low risk of bias. A total of 19 trials were considered at high risk of bias, and 9 at unclear risk of bias (Fig 2 and Figure A in S1 Appendix)

Studies results are reported in the text and in S1 Appendix.

### All-cause longest follow-up mortality

Overall, a total of 143/892 (16%) patients assigned to the furosemide bolus group died, as compared with 141/881 (16%) assigned to the control group, with no difference between the two groups (OR = 0.84; 95% CI 0.63 to 1.13; p-value = 0.25; I^2^ = 0%) (Table 2) (Figure B in S1 Appendix). Trial sequential analysis was inconclusive with only 25.79% of the information size accrued, suggesting the need for more randomized controlled trial(s) to establish firm evidence on the beneficial or detrimental effect on survival of furosemide bolus over control (OR 0.84, TSA-adjusted 95% CI, 0.46, 1.54) (Figure C in S1 Appendix). In particular, TSA estimated that the required information size would be 6874 randomized patients to show a 15% relative risk reduction.

**Table 2:** Overall results.

Analysis	Treatment group	Control group	OR/MD	95% CI	p-value for effect	p-value for heterogeneity	I^2^ (%)
Longest f-up mortality, n–events/N (%)	143/892 (16%)	141/881 (16%)	0.84	0.63 to 1.13	0.25	0.47	0
28/30 days mortality–events/N (%)	16/282 (8.8%)	11/312 (3.5%)	1.62	0.78 to 3.35	0.20	0.27	23
New/worsening AKI–events/N (%)	179/1335 (13.4%)	243/1333 (18.2%)	0.72	0.47 to 1.10	0.13	**0.001**	60
Need for RRT–events/N (%)	78/843 (9.3%)	94/842 (11.6%)	0.49	0.21 to 1.15	0.10	0.15	32
Hospital LOS, days–mean ± SD			0.17	-1.04 to 1.39	0.78	**0.003**	70
Peak serum creatinine, mg/dl			0.10	-0.12 to 0.33	0.36	**< 0.001**	98

CI: confidence interval; MD: mean difference; OR: odds ratio; AKI: acute kidney injury; RRT: renal replacement therapy; LOS, length of hospital stay.

**Fig 3:**
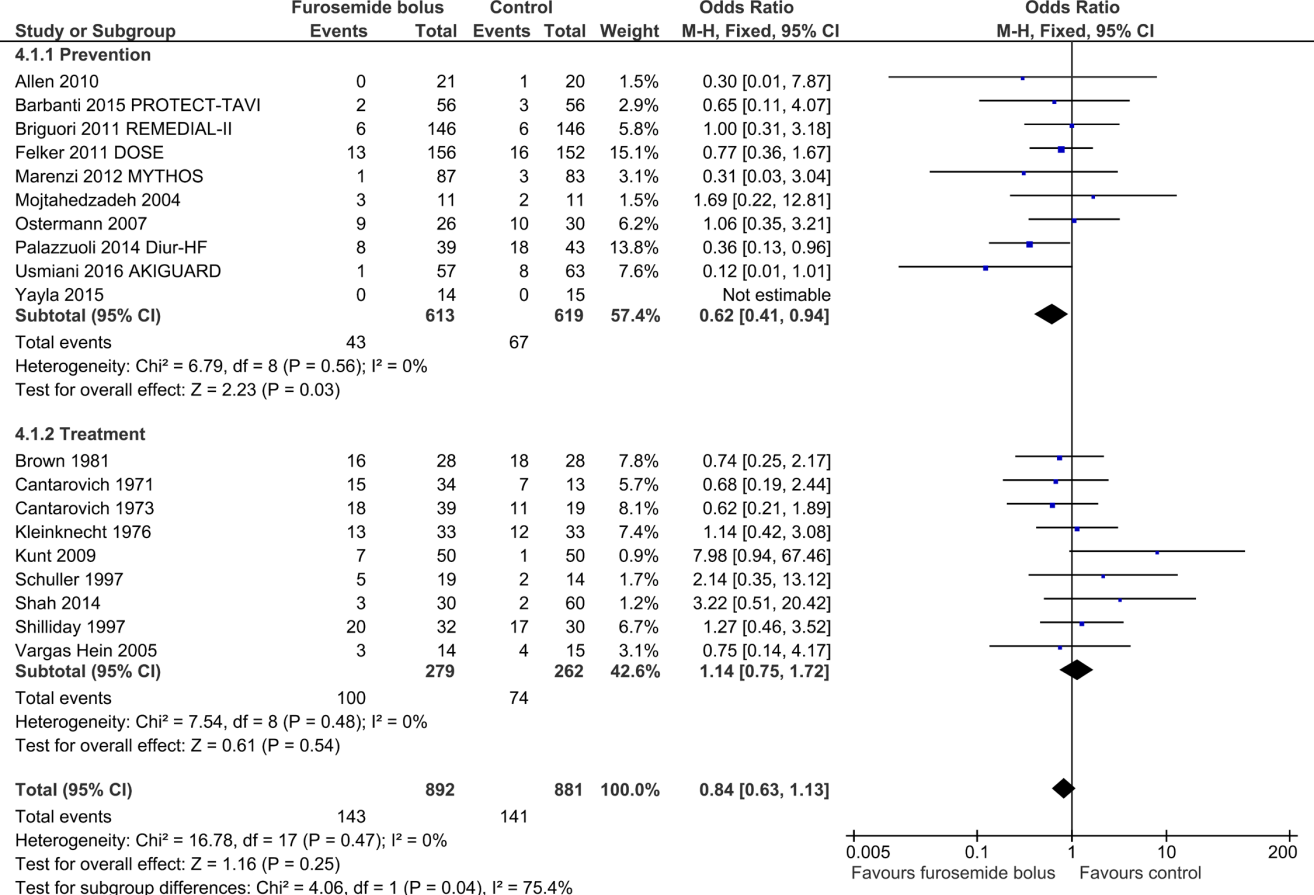
Furosemide prevention VS treatment subgroup.

A subgroup effect was identified only for studies administrating furosemide as prevention versus treatment, with a favorable effect in “prevention” trials (OR = 0.62; 95% CI 0.41 to 0.94; p-value = 0.03; I^2^ = 0%, with 9 studies included), and a neutral effect in “treatment” trials (OR = 1.14; 95% CI 0.75 to 1.72; p-value = 0.54; I^2^ = 0%, with 9 studies included), with a p-value between groups = 0.04. (Fig 3). Conversely, a subgroup effect depending on control treatment or clinical setting was not identified (Figures D and E in S1 Appendix). Trend towards subgroup differences were identified when stratifying analysis by control treatment and treatment indication (prevention vs treatment) (Figure D and Table D in S1 Appendix).

Sequentially removing each trial did not change magnitude and direction of treatment effect (lowest OR = 0.78; 95% CI 0.58 to 1.05; p-value = 0.10; I^2^ = 0%, removing Kunt et al [45]; highest OR = 0.90; 95% CI = 0.67 to 1.21; p-value = 0.49; I^2^ = 0%, removing Usmiani et al [55]).

As no trial with low risk of bias was identified, pre-specified analysis including only low risk of bias trials was not performed.

**Fig 4:**
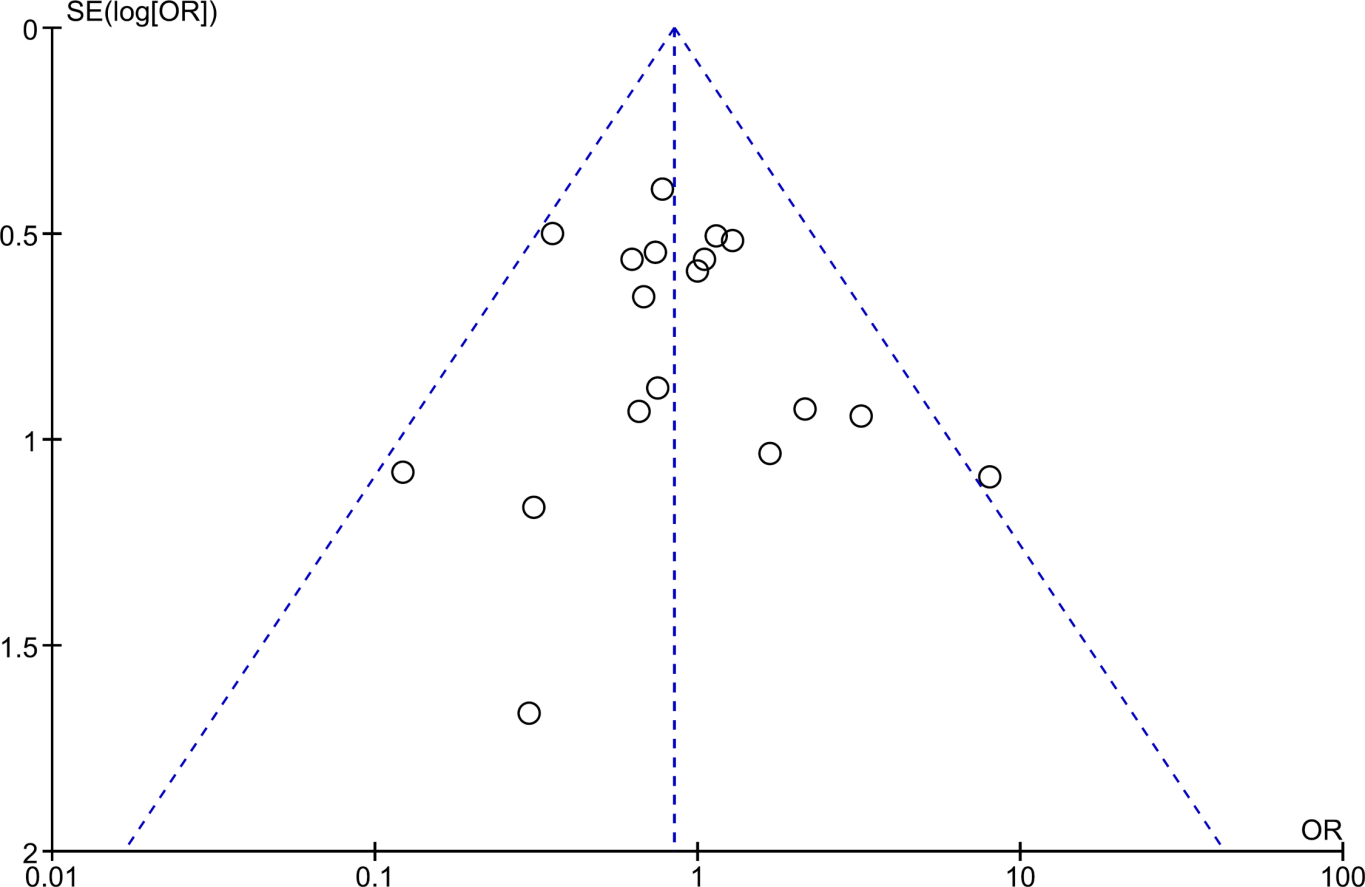
Funnel plot.

Visual inspection of funnel plot did not suggest possible presence of publication bias (Fig 4).

### All-cause 28/30-days mortality

Overall, we found no difference in 28/30 days mortality between the treatment and the control group (16/282 [5.7%] in the furosemide bolus group versus 11/312 [3.5%] in the control group; OR = 1.62; 95% CI 0.78 to 3.35; p-value = 0.20; I^2^ = 23%, with 4 studies included) (Figures F to I in S1 Appendix).

### New onset/worsening AKI

Overall, 179/1335 (13.4%) patients assigned to furosemide bolus administration experienced new onset or worsening of AKI, as compared with 243/1333 (18.2%) patients assigned to the control group (OR = 0.72; 95% CI = 0.47 to 1.10; p-value = 0.13; I^2^ = 60%, TSA inconclusive) (Figure J in S1 Appendix).

A subgroup effect was identified when analyzing trials according to the control treatment, with a favorable effect of furosemide bolus as compared with placebo/standard treatment and a harmful effect when compared with active pharmacological control (Figures K to M in S1 Appendix).

### Receipt of RRT

Overall, 78/843 (9.3%) patients assigned to furosemide bolus administration received RRT, as compared with 94/842 (11.6%) patients assigned to the control group (OR = 0.49; 95% CI = 0.21 to 1.15; p-value = 0.10; I^2^ = 32%, TSA inconclusive) (Figure N in S1 Appendix).

A trend towards a subgroup effect was identified when analyzing trials according to setting, with a favorable effect of furosemide bolus in the setting of contrast-induced AKI and a trend towards a harmful effect in cardiac surgery (Figure P in S1 Appendix). In addition, in the subgroup of patients receiving preventive furosemide administration, we observed a significant reduction in receipt of RRT (Figure Q in S1 Appendix).

### Length of hospital stay

Pooled analysis of the 6 trials that reported hospital length of stay did not show a statistically significant difference between furosemide bolus and control treatment (WMD = 0.17; 95% CI = -1.04 to 1.39; p-value = 0.78; I^2^ = 70%) (Figure R in S1 Appendix). A trend towards a subgroup difference was identified when comparing trials administering furosemide as a preventive measure as compared with trials administering it as treatment (Figures S to U in S1 Appendix).

### Peak serum creatinine

Pooled analysis of the 6 trials reporting peak serum creatinine after randomization did not show a statistically significant difference between furosemide bolus and control treatment (WMD = 0.10; 95% CI = -0.12 to 0.33; p-value = 0.36; I^2^ = 98%) (Figure V in S1 Appendix). A subgroup effect was identified when analyzing trials according to the setting, with trials performed in cardiac surgery setting showing an increase in serum creatinine associated with bolus furosemide administration (Figures V to Y in S1 Appendix).

### Contrast-induced AKI

Subgroup analyses comparing trials performed in the setting of contrast-induced AKI versus trials performed in all other settings did not identify a subgroup effect except in any of the analysed outcomes with the exception of all-cause 28/30-days mortality (p for interaction = 0.04) (Figures AB to AF in S1 Appendix).

## Discussion

### Key findings

In this meta-analysis of RCTs, we found that intermittent furosemide administration in patients with or at risk for AKI did not result in a lower mortality, reduced incidence or worsening of AKI, or decreased utilization of RRT. A trend towards a beneficial effect of intermittent furosemide administration was found when analyzing the subgroup of studies in which furosemide was administered to prevent AKI. This finding was consistent across different outcomes, with a similar beneficial effect on RRT utilization and a trend towards a beneficial effect on worsening AKI, length of hospital stay, and peak serum creatinine in “prevention” trials. However, TSA was inconclusive, suggesting no firm conclusions on the topic and the need of further high-quality studies on the topic.

### Relationship to previous studies

The effect of loop diuretics on incidence and course of AKI has been a matter of debate and investigation for years. Accordingly, several RCTs and observational trials have been performed investigating the effect of diuretics and optimal diuretic strategy. As of today, convincing evidence that diuretic administration can alter *per se* the course of AKI or shorten renal recovery when RRT is needed is lacking [4,58,59].

In the largest meta-analysis performed so far on furosemide administration in patients with or at risk for AKI, the authors included a total of 11 studies and found no difference in mortality or RRT utilization between the furosemide and control group, for both AKI prevention and treatment [60]. Compared with this study, our meta-analysis specifically investigated the role of intermittent furosemide administration versus any comparator, including continuous furosemide infusion. In addition, we included a larger number of trials and patients, including some recent, large multicenter RCTs [42,46].

Other meta-analyses on the role of loop diuretics in prevention or treatment of AKI in different settings and with different inclusion criteria have been performed [61,62,63,64], all of which consistently found that loops diuretics administration was not associated with improved outcome in patients with AKI. Meta-analyses consistently confirmed a higher urine output associated with diuretics use [62,63] and some suggested a possible shorter duration of RRT [62] and number of dialysis treatments [63], although level of evidence was considered to be low. A positive effect of furosemide administration on RRT use was confirmed in our study, although limited to trials investigating preventive role of furosemide. Conversely, meta-analyses and small trials questioned the beneficial effect of furosemide on incidence or clinical course of AKI [37,61,65]. To clarify these issues, a pilot multicenter RCT on continuous furosemide administration versus placebo in critically ill patients with AKI (the SPARK study) was planned [66]. The study aimed at enrolling 216 patients with early AKI defined according to R-RIFLE criteria [67], with worsening AKI as primary endpoint. Unfortunately, the trial was interrupted early due to logistic problems and lack of funding after only 73 patients had been enrolled, and found no difference in the primary or any of secondary outcomes [68]. However, furosemide administration was associated with a higher risk of electrolyte abnormalities.

Several meta-analyses compared continuous versus intermittent furosemide administration in patients several clinical settings, both in adults and pediatric patients [69,70,71,72]. This meta-analyses yielded small differences in results depending on trial inclusion criteria, but consistently confirmed no improvement in major outcomes associated with any of the two strategies. The largest meta-analysis, including all RCTs performed on hospitalized patients (including pediatric and crossover studies), suggested that continuous administration might be associated with greater urine output [72], especially when preceded by a single bolus. Whether this translates in improved clinical outcome remains to be determined [73]. Compared with previously published meta-analyses, we focused on adult patients only and included a larger number of trials and comparators, while excluding crossover studies, as our meta-analysis aimed at investigating clinically relevant outcomes such as mortality.

### Significance of study findings

Our study suggested that furosemide administration may have some benefits when used to prevent AKI, both in terms of mortality and need for RRT. However, we believe that our results should be interpreted with caution, as also suggested by TSA that was inconclusive due to too low information size. Indeed, positive results observed with furosemide administration are largely driven by trials performed in the setting of CI-AKI, and in particular by four trials investigating an automated fluid delivery system (RenalGuard, PLC Medical Systems, Milford, Massachusetts) which matches hydration with diuresis [41,42,50,58]. In these studies, furosemide administration to maintain a diuresis ≥ 300 mL/h with matched hydration was shown to prevent CI-AKI and subsequent need for RRT without major adverse events related to fluid overload [74]. Notably, volume expansion is currently the only widely recommended strategy to prevent CI-AKI [75,76] while furosemide was shown to have detrimental effect when not coupled with adequate hydration [22,23,45,77]. Accordingly, beneficial effect of RenalGuard system is probably related not on furosemide administration, but on the adequate diuresis achieved together with targeted volume expansion, thus limiting the risk of both fluid overload and dehydration. To further complicate the picture, published RCTs on RenalGuard system were all considered to be at high risk of bias, thereby downgrading level of evidence of this strategy [74], while the effectiveness of volume expansion in preventing CI-AKI has been recently challenged [78].

The role of fluid overload on incidence and pathogenesis of AKI and organ dysfunction in critically ill and surgical patients has been largely investigated in recent years [19,79,80,81]. It is now generally recognized that also excessive fluid overload and high central venous pressure causes renal congestion and impaired kidney perfusion, both in heart failure and critically ill patients. In this context, positive effects observed with furosemide administration in patients with AKI may be related to an indirect mechanism mediated by fluid removal, rather than a direct positive effect of loop diuretics. Accordingly, recent guidelines recommend diuretics use to optimize patient volume status [4,35]. A major problem in fluid management in patients with AKI is that both hypovolemia and hypervolemia are associated with AKI development and progression [79,80]. As a consequence, the same diuretic that might improve renal function in fluid-overloaded patient may have detrimental effect on kidney perfusion if a patient is or become volume-depleted. This dual effect can explain the controversial results obtained so far by studies investigating the role of diuretics in prevention or treatment of AKI.

It is a common clinical observation that reversal of oligo-anuria with furosemide administration is frequently associated with improvement in renal function. A furosemide stress test to assess AKI severity and likelihood of progression has been recently developed and validated [82,83]. Chawla et al. showed that a diuresis of at least 200 mL in two hours following a 1–1.5 mg/kg furosemide bolus was associated with reduced AKI progression. This might occur because adequate diuretic response to furosemide required adequate renal perfusion, active secretion of the drug in the tubule, and absence of urinary flow obstruction, all indicative of less severe injury and adequate renal reserve. Thereby, this simple test could globally evaluate kidney function and renal reserve. Therefore, in clinical practice, reversal of oligo-anuria with furosemide is more likely a marker of reduced kidney injury and/or dysfunction, rather than of a beneficial effect exerted by furosemide.

### Limitations of the study

Our study has some limitations, which are characteristics of all aggregate data meta-analyses [24,84]. First, we included several studies performed in different setting, with different aims and different control groups. However, we also performed several subgroup analyses, which helped us to better define the influence of each subgroup on overall results. Second, all of the included trials were considered to carry an unclear or a high risk of bias, thereby reducing quality of evidence that our meta-analysis can provide. Nevertheless, identifying lack of high-quality trials and gaps in evidence on a topic is also an objective of systematic reviews and meta-analyses. In a similar context, meta-analyses should be considered hypothesis-generating rather than confirmatory. Third, some of the included studies were performed decades ago. Finally, we focused on adult patients receiving intermittent furosemide administration. Therefore, our results may not apply to continuous furosemide administration or pediatric population, although previous meta-analyses did not suggest that a different effect in this setting is expected.

### Future studies and prospects

The role of furosemide and, diuretics administration in patients with or at risk for AKI still needs to be clearly determined. While it is generally accepted that available diuretics are unlikely to exert a direct kidney-protective effect in real-world clinical practice, an indirect effect through optimization of volume status can not be excluded. Future trials should better address this issue. An ideal trial should investigate whether optimization of volume status with diuretics reduces AKI development or progression. However, such a study is unlikely to be conducted mainly due to organizing and ethical reasons. Alternatively, it may be interesting to compare a diuretic-based versus an early-RRT-based fluid management strategy in AKI.

## Conclusions

Randomized trials showed that intermittent furosemide administration is not associated with an overall improvement in survival or other major outcomes in patients with or at risk for AKI, although it may reduce mortality and RRT utilization when used as a preventive measure. However, these findings are largely influenced by a specific subset of trials performed in CI-AKI setting and likely attributable to the concomitant management protocol investigated in these trials. Furthermore, low risk of bias trials are lacking. Future high quality trials are needed to confirm the role of loop diuretics in AKI prevention and management, although ethical issues may limit feasibility.

## Supporting information

S1 Checklist PRISMA Checklist. Checklist for transparent and complete reporting of systematic reviews and meta-analyses.
(PDF)

S1 Appendix Supplementary appendix. Supplementary Appendix including.
(DOCX)
