# Supplementary material for: Intermittent furosemide administration in patients with or at risk for acute kidney injury: Meta-analysis of randomized trials
Source: PLoS One. 2018 Apr 24;13(4):e0196088. doi: 10.1371/journal.pone.0196088 (PMC5915682; doi:10.1371/journal.pone.0196088)
Supplement: S1 Appendix — Supplementary Appendix including. (DOCX) [file pone.0196088.s002.docx]

**S1 APPENDIX FILE**

**SUPPLEMENTARY APPENDIX**

Intermittent furosemide administration in patients with or at risk for acute kidney injury: meta-analysis of randomized trials

*T. Bove, A. Belletti, A. Putzu, S. Pappacena,*

*G. Denaro, G. Landoni, S.M. Bagshaw, A. Zangrillo*

**TABLE OF CONTENTS**

**Search strategy for PubMed**

**Supplementary Table A –** List of excluded studies and reasons

**Supplementary Table B -** Analysis by control treatment

**Supplementary Table C -** Analysis by setting

**Supplementary Table D –** Analysis by prevention vs treatment

**Supplementary Figure A -** Summary of risk of bias assessment

**Supplementary Figure B -** Forest plot for all-cause mortality

**Supplementary Figure C -** Trial sequential analysis for mortality

**Supplementary Figure D -** Forest plot for all-cause mortality, by control treatment

**Supplementary Figure E -** Forest plot for all-cause mortality, by setting

**Supplementary Figure F -** Forest plot for 28/30-days mortality

**Supplementary Figure G -** Forest plot for 28/30-days mortality, by control treatment

**Supplementary Figure H -** Forest plot for 28/30-days mortality, by setting

**Supplementary Figure I -** Forest plot for 28/30-days mortality, by treatment indication

**Supplementary Figure J -** Forest plot for AKI

**Supplementary Figure K -** Forest plot for AKI, by control treatment

**Supplementary Figure L -** Forest plot for AKI, by setting

**Supplementary Figure M -** Forest plot for AKI, by treatment indication

**Supplementary Figure N –** Forest plot for receipt of RRT

**Supplementary Figure O -** Forest plot for receipt of RRT, by control treatment

**Supplementary Figure P -** Forest plot for receipt of RRT, by setting

**Supplementary Figure Q -** Forest plot for receipt of RRT, by treatment indication

**Supplementary Figure R -** Forest plot for length of hospital stay

**Supplementary Figure S -** Forest plot for length of hospital stay, by control treatment

**Supplementary Figure T -** Forest plot for length of hospital stay, by setting

**Supplementary Figure U -** Forest plot for length of hospital stay, by treatment indication

**Supplementary Figure V** Forest plot for peak serum creatinine

**Supplementary Figure W -** Forest plot for peak serum creatinine, by control treatment

**Supplementary Figure X -** Forest plot for peak serum creatinine, by setting

**Supplementary Figure Y -** Forest plot for peak serum creatinine, by treatment indication

**Supplementary Figure Z -** Forest plot for all-cause mortality, contrast-induced AKI versus non contrast-induced AKI

**Supplementary Figure AB -** Forest plot for 28/30 days all-cause mortality, contrast-induced AKI versus non contrast-induced AKI

**Supplementary Figure AC -** Forest plot for AKI, contrast-induced AKI versus non contrast-induced AKI

**Supplementary Figure AD -** Forest plot for receipt of renal-replacement therapy, contrast-induced AKI versus non contrast-induced AKI

**Supplementary Figure AE -** Forest plot for hospital length of stay, contrast-induced AKI versus non contrast-induced AKI

**Supplementary Figure AF -** Forest plot for peak serum creatinine, contrast-induced AKI versus non contrast-induced AKI

**Supplementary References**

**Search strategy for PubMed**

(furosemide OR frusemide) AND (randomized controlled trial[pt] OR controlled clinical trial[pt] OR randomized controlled trials[mh] OR random allocation[mh] OR double-blind method[mh] OR single-blind method[mh] OR clinical trial[pt] OR clinical trials[mh] OR (clinical trial[tw] OR ((singl*[tw] OR doubl*[tw] OR trebl*[tw] OR tripl*[tw]) AND (mask*[tw] OR blind[tw])) OR (latin square[tw]) OR placebos[mh] OR placebo*[tw] OR random*[tw] OR research design[mh:noexp] OR follow-up studies[mh] OR prospective studies[mh] OR cross-over studies[mh] OR control*[tw] OR prospectiv*[tw] OR volunteer*[tw]) NOT (animal[mh] NOT human[mh]) NOT (comment[pt] OR editorial[pt] OR meta-analysis[pt] OR practice-guideline[pt] OR review[pt]))

**Supplementary Table A.** List of excluded studies, together with reason for exclusion

| **First Author** | **Year** | **Journal** | **Reason for exclusion** |
| --- | --- | --- | --- |
| Badawy S | 2012 | *J Crit Care* | Continuous furosemide infusion |
| Bagshaw SM | 2017 | *J Crit Care* | Continuous furosemide infusion |
| Bagshaw SM | 2010 | *Trials* | Study protocol paper |
| Bart BA | 2005 | *J Am Coll Cardiol* | No bolus furosemide group |
| Bart BA | 2012 | *N Engl J Med* | No bolus furosemide group |
| Cantarovich F | 2004 | *Am J Kidney Dis* | Continuous furosemide infusion |
| Costanzo MR | 2007 | *J Am Coll Cardiol* | No bolus furosemide group |
| Costanzo MR | 2010 | *J Card Fail* | Overlapping population |
| Fakhari S | 2017 | *Res Rep Urol* | Continuous furosemide infusion |
| Cotter G | 1997 | *Clin Pharmacol Ther* | Oral furosemide |
| Giglioli C | 2011 | *Eur J Heart Fail* | No outcome data |
| Glamouzis G | 2010 | *J Card Fail* | Continuous furosemide infusion |
| Hager B | 1996 | *Schweiz Med Wochenschr* | Continuous furosemide infusion |
| Hamishekhar H | 2017 | *Indian J Crit Care Med* | Continuous furosemide infusion |
| Hanna MA | 2012 | *Congest Heart Fail* | No bolus furosemide group |
| Khan NA | 2010 | *Am J Med* | Oral furosemide |
| Klinge JM | 1997 | *Intensive Care Med* | Pediatric population |
| Kwiatkowski DM | 2017 | *JAMA Pediatr* | Pediatric population |
| Lassnigg A | 2000 | *J Am Soc Nephrol* | Continuous furosemide infusion |
| Licata G | 2003 | *Am Heart J* | Overlapping population |
| Lim E | 2002 | *Ann Thorac Surg* | Oral furosemide |
| Luciani GB | 1997 | *Ann Thorac Surg* | Pediatric population |
| Majumdar SR | 2009 | *Am J Kidney Dis* | Continuous furosemide infusion |
| Malfatto G | 2012 | *J Cardiovasc Pharmacol* | Continuous furosemide infusion |
| Nuutinen L | 1976 | *Ann Surg Gynaecol* | Non-randomized design |
| Nuutinen L | 1978 | *J Cardiovasc Surg* | Non-randomized design |
| Palazzuoli A | 2015 | *Intern Emerg Med* | Overlapping population |
| Paterna S | 2000 | *Eur J Heart Fail* | Inadequate control group |
| Rogers HL | 2008 | *J Card Fail* | Overlapping population |
| Shemirani H | 2012 | *Saudi J Kidney Dis Transpl* | Oral furosemide |
| Singh NC | 1992 | *Crit Care Med* | Pediatric population |
| Sirivella S | 2000 | *Ann Thorac Surg* | No bolus furosemide group |
| Triposkiadis F | 2014 | *Int J Cardiol* | Continuous furosemide infusion |
| Van der Voort PH | 2009 | *Crit Care Med* | Continuous furosemide infusion |
| Weisberg LS | 1994 | *Kidney Int* | No bolus furosemide group |

**Supplementary Table B – Analysis by control treatment**

| **Analysis** | **Treatment group** | **Control group** | **OR/MD** | **95% CI** | **p-value for effect** | **p-value for heterogeneity** | **I^2^ (%)** | **p-value for subgroup differences** |
| --- | --- | --- | --- | --- | --- | --- | --- | --- |
| Longest f-up mortality, n – events/N (%) | 143/892 (16%) | 141/881 (16%) | 0.84 | 0.63 to 1.13 | 0.25 | 0.47 | 0 | 0.84 |
| - Furosemide infusion | 64/394 (16.2%) | 70/423 (16.5%) | 0.98 | 0.57 to 1.69 | 0.95 | 0.19 | 29 |  |
| - Placebo/standard treatment | 76/484 (15.7%) | 67/443 (15.1%) | 0.80 | 0.51 to 1.26 | 0.34 | 0.60 | 0 |  |
| - Active pharmacological control | 3/14 (21.4%) | 4/15 (26.6%) | 0.75 | 0.14 to 4.17 | 0.74 | N/A | N/A |  |
| 28/30 days mortality – events/N (%) | 16/282 (8.8%) | 11/312 (3.5%) | 1.55 | 0.56 to 4.28 | 0.40 | 0.26 | 23 | 0.08 |
| - Furosemide infusion | 8/80 (10.0%) | 2/60 (3.3%) | 4.71 | 0.99 to 22.45 | 0.05 | 0.43 | 0 |  |
| - Placebo/standard treatment | 8/202 (4.0%) | 11/312 (3.5%) | 0.89 | 0.33 to 2.35 | 0.81 | 0.70 | 0 |  |
| New/worsening AKI – events/N (%) | 179/1335 (13.4%) | 243/1333 (18.2%) | 0.72 | 0.47 to 1.10 | 0.13 | 0.001 | 78% | 0.01 |
| - Furosemide infusion | 55/388 (14.1%) | 49/352 (13.9%) | 0.93 | 0.60 to 1.43 | 0.73 | 0.50 | 0 |  |
| - Placebo/standard treatment | 114/922 (12.4%) | 184/928 (19.8%) | 0.49 | 0.28 to 0.85 | 0.01 | 0.01 | 60 |  |
| - Active pharmacological control | 10/25 (40%) | 10/53 (18.9%) | 2.87 | 1.00 to 8.24 | 0.05 | N/A | N/A |  |
| Need for RRT – events/N (%) | 78/843 (9.6%) | 94/842 (11.2%) | 0.49 | 0.21 to 1.15 | 0.10 | 0.15 | 38.1% | 0.20 |
| - Furosemide infusion | 34/343 | 32/344 | 1.39 | 0.21 to 9.28 | 0.73 | 0.13 | 48 |  |
| - Placebo/standard treatment | 44/500 (8.8%) | 62/498 (12.4%) | 0.36 | 0.15 to 0.85 | 0.02 | 0.31 | 16 |  |
| Hospital LOS, days – mean ± SD |  |  | 0.17 | -1.04 to 1.39 | 0.78 | 0.003 | 70 | 0.71 |
| - Furosemide infusion |  |  | 0.09 | -1.39 to 1.58 | 0.90 | 0.001 | 75 |  |
| - Placebo/standard treatment |  |  | 0.50 | -1.08 to 2.08 | 0.54 | N/A | N/A |  |
| Peak serum creatinine |  |  | 0.10 | -0.12 to 0.33 | 0.36 | < 0.001 | 98 | 0-64 |
| - Furosemide infusion |  |  | 0.07 | -0.56 to 0.69 | 0.83 | < 0.001 | 97 |  |
| - Placebo/standard treatment |  |  | 0.14 | -0.16 to 0.45 | 0.35 | < 0.001 | 99 |  |
| - Active pharmacological treatment |  |  | 0.72 | -0.50 to 1.94 | 0.25 | N/A | N/A |  |

**Supplementary Table C – Analysis by setting**

| **Analysis** | **Treatment group** | **Control group** | **OR/MD** | **95% CI** | **p-value for effect** | **p-value for heterogeneity** | **I^2^ (%)** | **p-value for subgroup differences** |
| --- | --- | --- | --- | --- | --- | --- | --- | --- |
| Longest f-up mortality, n – events/N (%) | 143/887 (16.1%) | 141/886 (15.9%) | 0.87 | 0.62 to 1.21 | 0.41 | 0.32 | 0 | 0.47 |
| - AKI/ICU | 99/217 (45.6%) | 79/183 (43.2%) | 1.02 | 0.67 to 1.56 | 0.92 | 0.70 | 0 |  |
| - Heart Failure | 24/260 (9.2%) | 37/290 (12.8%) | 0.70 | 0.31 to 1.57 | 0.38 | 0.20 | 36 |  |
| - Cardiac Surgery | 10/64 (15.6%) | 5/65 (8.0%) | 2.24 | 0.21 to 23.53 | 0.50 | 0.09 | 66 |  |
| - Contrast-induced AKI | 10/346 (2.9%) | 20/348 (5.7%) | 0.50 | 0.23 to 1.08 | 0.08 | 0.34 | 10 |  |
| - Non- contrast-induced AKI | 133/546 (%) | 121/533 (%) | 0.92 | 0.67 to 1.27 | 0.63 | 0.48 | 0 | 0.14* |
| 28/30 days mortality – events/N (%) | 16/282 (5.7%) | 11/312 (3.5%) | 1.55 | 0.56 to 4.28 | 0.40 | 0.26 | 46.4% | 0.15 |
| - Heart Failure | 3/30 (10.0%) | 2/60 (3.3%) | 3.22 | 0.51 to 20.42 | 0.21 | N/A | N/A |  |
| - Cardiac Surgery | 5/50 (10.0%) | 0/50 (0.0%) | 12.21 | 0.66 to 226.97 | 0.09 | N/A | N/A |  |
| - Contrast-induced AKI | 8/202 (4.0%) | 9/202 (4.5%) | 0.88 | 0.33 to 2.34 | 0.80 | 0.70 | 0 |  |
| - Non- contrast-induced AKI | 8/80 (%) | 2/110 (%) | 5.66 | 1.25 to 25.57 | 0.02 | 0.43 | 0 | 0.04* |
| New/worsening AKI – events/N (%) | 179/1335 (13.4%) | 243/1333 (18.2%) | 0.72 | 0.47 to 1.10 | 0.13 | 0.001 | 30.2% | 0.23 |
| - AKI/ICU | 42/91 (46.2%) | 43/93 (46.2%) | 0.83 | 0.34 to 2.03 | 0.69 | 0.62 | 0 |  |
| - Heart Failure | 49/312 (15.7%) | 49/272 (18.0%) | 0.85 | 0.55 to 1.32 | 0.48 | 0.93 | 0 |  |
| - Cardiac Surgery | 5/50 (10.0%) | 0/50 (0.0%) | 12.21 | 0.66 to 226.97 | 0.09 | N/A | N/A |  |
| - Contrast-induced AKI | 83/882 (9.4%) | 151/918 (16.4%) | 0.57 | 0.28 to 1.16 | 0.12 | 0.0001 |  |  |
| - Non- contrast-induced AKI | 96/453 (%) | 92/415 (%) | 0.89 | 0.60 to 1.32 | 0.56 | 0.70 | 0 | 0.28* |
| Need for RRT – events/N (%) | 78/843 (9.3%) | 94/842 (11.2%) | 0.49 | 0.21 to 1.15 | 0.10 | 0.15 | 57.6 | 0.07 |
| - AKI/ICU | 70/129 (54.3%) | 77/131 (58.8%) | 0.61 | 0.20 to 1.82 | 0.37 | 0.23 | 29 |  |
| - Heart Failure | 1/239 (0.4%) | 5/236 (2.1%) | 0.19 | 0.02 to 1.64 | 0.13 | N/A | N/A |  |
| - Cardiac Surgery | 5/50 (10.0%) | 0/50 (0.0%) | 12.21 | 0.66 to 226.97 | 0.09 | N/A | N/A |  |
| - Contrast-induced AKI | 2/425 (0.5%) | 12/425 (2.8%) | 0.20 | 0.05 to 0.81 | 0.02 | 0.87 | 0 |  |
| - Non- contrast-induced AKI | 76/418 (%) | 82/417 (%) | 0.69 | 0.23 to 2.10 | 0.51 | 0.09 | 45 | 0.18* |
| Hospital LOS, days – mean ± SD |  |  | 0.17 | -1.04 to 1.39 | 0.78 | 0.003 | 70 | 0.36 |
| - Heart Failure |  |  | -0.25 | -2.28 to 1.78 | 0.81 | 0.005 | 73 |  |
| - Cardiac Surgery |  |  | 1.26 | 0.31 to 2.21 | 0.01 | N/A | N/A |  |
| - Contrast-induced AKI |  |  | 0.50 | -1.08 to 2.08 | 0.54 | N/A | N/A |  |
| - Non- contrast-induced AKI |  |  | 0.09 | -1.39 to 1.58 | 0.90 | 0.001 | 75 | 0.71* |
| Peak serum creatinine |  |  | 0.10 | -0.12 to 0.33 | 0.36 | < 0.001 | 98 | 0.44 |
| - AKI/ICU |  |  | -0.22 | -0.32 to -0.12 | < 0.001 | N/A | N/A |  |
| - Heart Failure |  |  | -0.44 | -0.64 to -0.24 | < 0.001 | N/A | N/A |  |
| - Cardiac Surgery |  |  | 0.60 | 0.08 to 1.12 | 0.02 | < 0.001 | 90 |  |

* denotes p-value for subgroup differences between contrast-induced AKI trials and non- contrast-induced AKI trials.

**Supplementary Table D – Analysis by prevention vs treatment**

| **Analysis** | **Treatment group** | **Control group** | **OR/MD** | **95% CI** | **p-value for effect** | **p-value for heterogeneity** | **I^2^ (%)** | **p-value for subgroup differences** |
| --- | --- | --- | --- | --- | --- | --- | --- | --- |
| Longest f-up mortality, n – events/N (%) | 143/892 (16.0%) | 141/881 (16.0%) | 0.84 | 0.63 to 1.13 | 0.25 | 0.47 | 75.4 | 0.04 |
| - Prevention | 43/613 (7.0%) | 67/619 (10.8%) | 0.62 | 0.41 to 0.94 | 0.03 | 0.56 | 0 |  |
| - Treatment | 100/279 (35.8%) | 74/262 (28.2%) | 1.14 | 0.75 to 1.72 | 0.54 | 0.48 | 0 |  |
| 28/30 days mortality – events/N (%) | 16/282 (5.7%) | 11/312 (3.5%) | 1.55 | 0.56 to 4.28 | 0.40 | 0.26 | 68.4 | 0.08 |
| - Prevention | 8/202 (3.7%) | 9/202 (4.5%) | 0.89 | 0.33 to 2.35 | 0.81 | 0.70 | 0 |  |
| - Treatment | 8/80 (10.0%) | 2/110 (1.8%) | 4.71 | 0.99 to 22.45 | 0.05 | 0.43 | 0 |  |
| New/worsening AKI – events/N (%) | 179/1335 (13.4%) | 243/1333 (18.2%) | 0.72 | 0.47 to 1.10 | 0.13 | 0.001 | 0 | 0.44 |
| - Prevention | 128/1190 (10.8%) | 195/1194 (16.3%) | 0.66 | 0.40 to 1.08 | 0.10 | 0.0006 | 68 |  |
| - Treatment | 51/145 (35.2%) | 48/139 (34.5%) | 0.97 | 0.42 to 2.28 | 0.95 | 0.31 | 16 |  |
| Need for RRT – events/N (%) | 78/843 (9.3%) | 94/842 (11.2%) | 0.49 | 0.21 to 1.15 | 0.10 | 0.15 | 18.0 | 0.27 |
| - Prevention | 4/660 (0.6%) | 17/665 (2.6%) | 0.28 | 0.09 to 0.83 | 0.02 | 0.56 | 0 |  |
| - Treatment | 74/183 (40.4%) | 77/177 (43.5%) | 0.74 | 0.19 to 2.92 | 0.67 | 0.07 | 53 |  |
| Hospital LOS, days – mean ± SD |  |  | 0.17 | -1.04 to 1.39 | 0.78 | 0.003 | 70 | 0.06 |
| - Prevention |  |  | -0.61 | -1.96 to 0.73 | 0.37 | 0.05 | 58 |  |
| - Treatment |  |  | 2.16 | -0.36 to 4.68 | 0.09 | 0.12 | 59 |  |
| Peak serum creatinine |  |  | 0.10 | -0.12 to 0.33 | 0.36 | < 0.001 | 98 | < 0.001 |
| - Prevention |  |  | -0.08 | -0.31 to 0.15 | 0.52 | < 0.001 | 99 |  |
| - Treatment |  |  | 0.88 | 0.63 to 1.14 | < 0.001 | 0.79 | 0 |  |

**Supplementary Figure A.** Summary of risk of bias assessment for included studies.

**
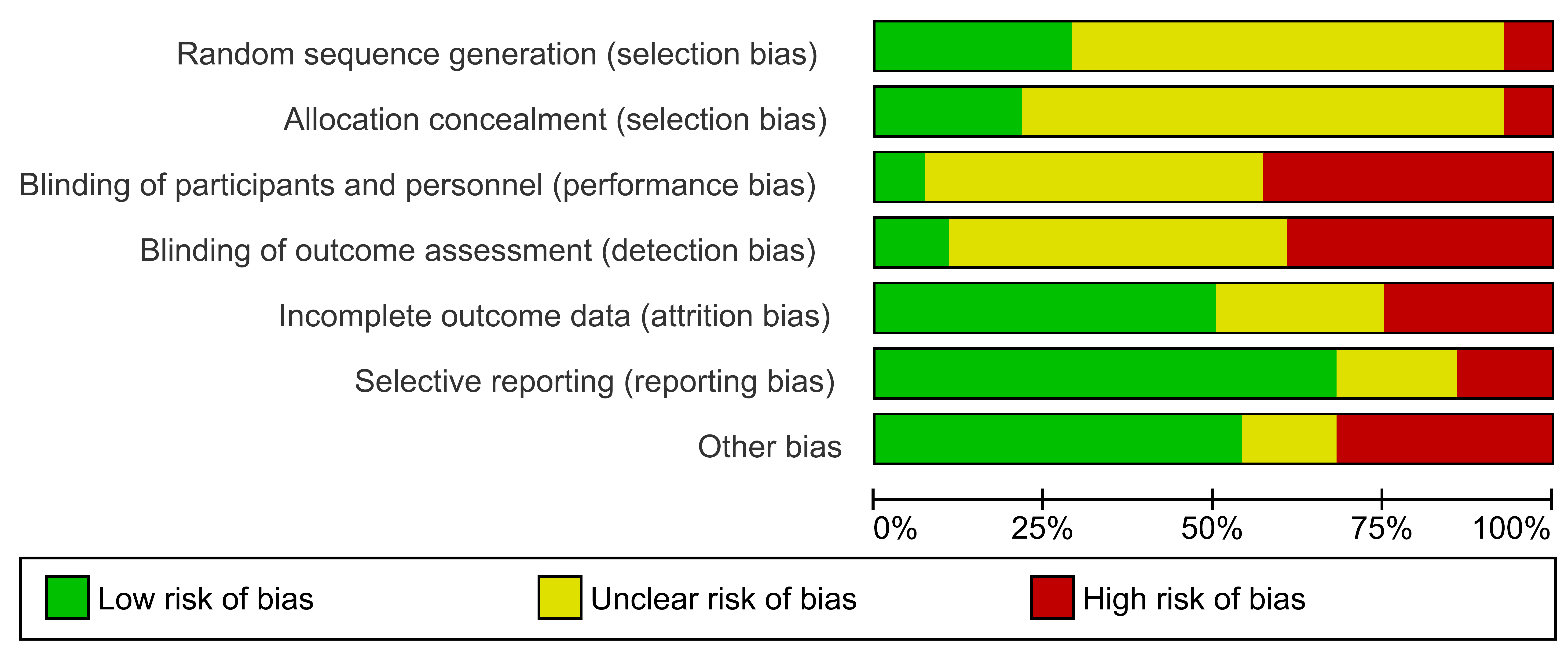
**

**Supplementary Figure B.** Forest plot for all-cause mortality at the longest follow-up available

**Supplementary Figure C.** Trial sequential analysis for mortality at longest follow-up available.

Trial sequential analysis of the randomized trials (black square fill icons) shows that the cumulative Z-curve did not cross the trial sequential monitoring boundary, suggesting the need for more trials to establish firm conclusions about bolus furosemide survival benefit or harm. X-axis: the number of patients randomized; Y-axis: the cumulative Z-Score; Horizontal dotted lines: conventional boundaries (two-sided, p value = 0.05); Oblique lines with diamond icons: trial sequential monitoring boundaries; Oblique line with square fill icons: Z-curve; Vertical line with diamonds: required information size.


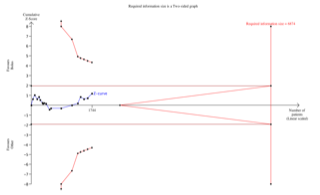


**Supplementary Figure D.** Forest plot for all-cause mortality at the longest follow-up available, stratified by control treatment

**Supplementary Figure E.** Forest plot for all-cause mortality at the longest follow-up available, stratified by setting

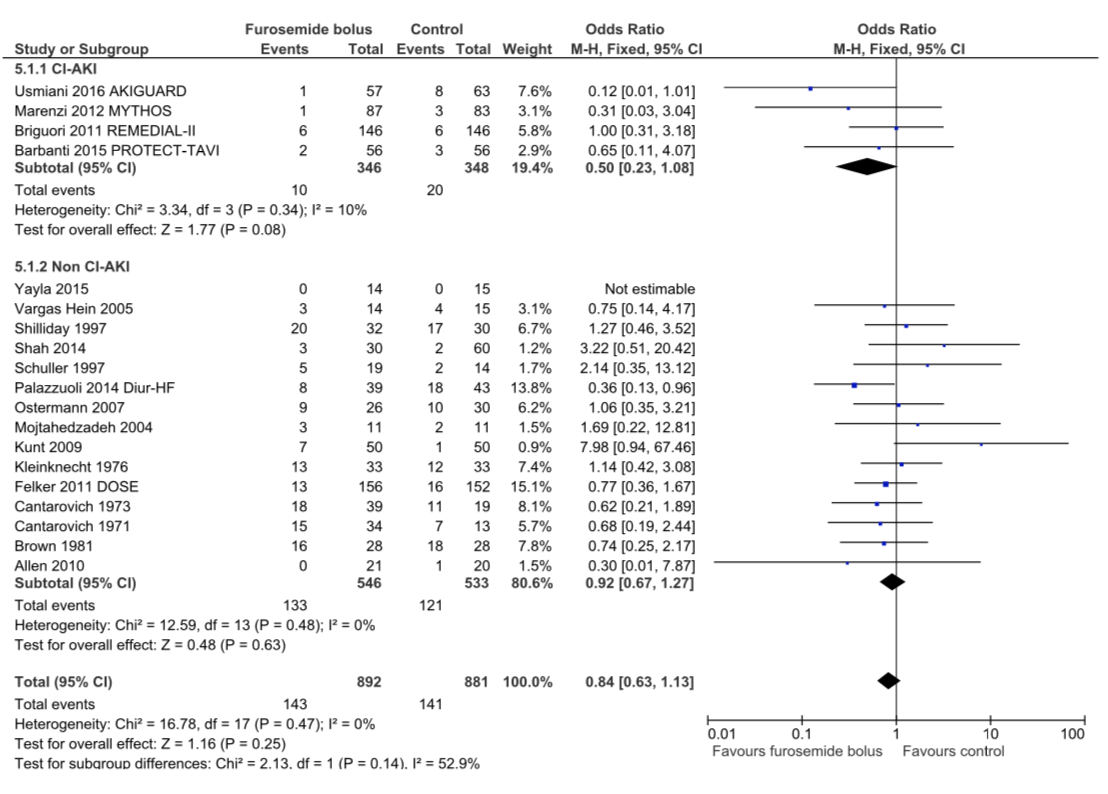


**Supplementary Figure F.** Forest plot for 28/30-days mortality at the longest follow-up available

**Supplementary Figure G.** Forest plot for 28/30-days mortality at the longest follow-up available, stratified by control treatment

**Supplementary Figure H.** Forest plot for 28/30-days mortality at the longest follow-up available, stratified by setting

**
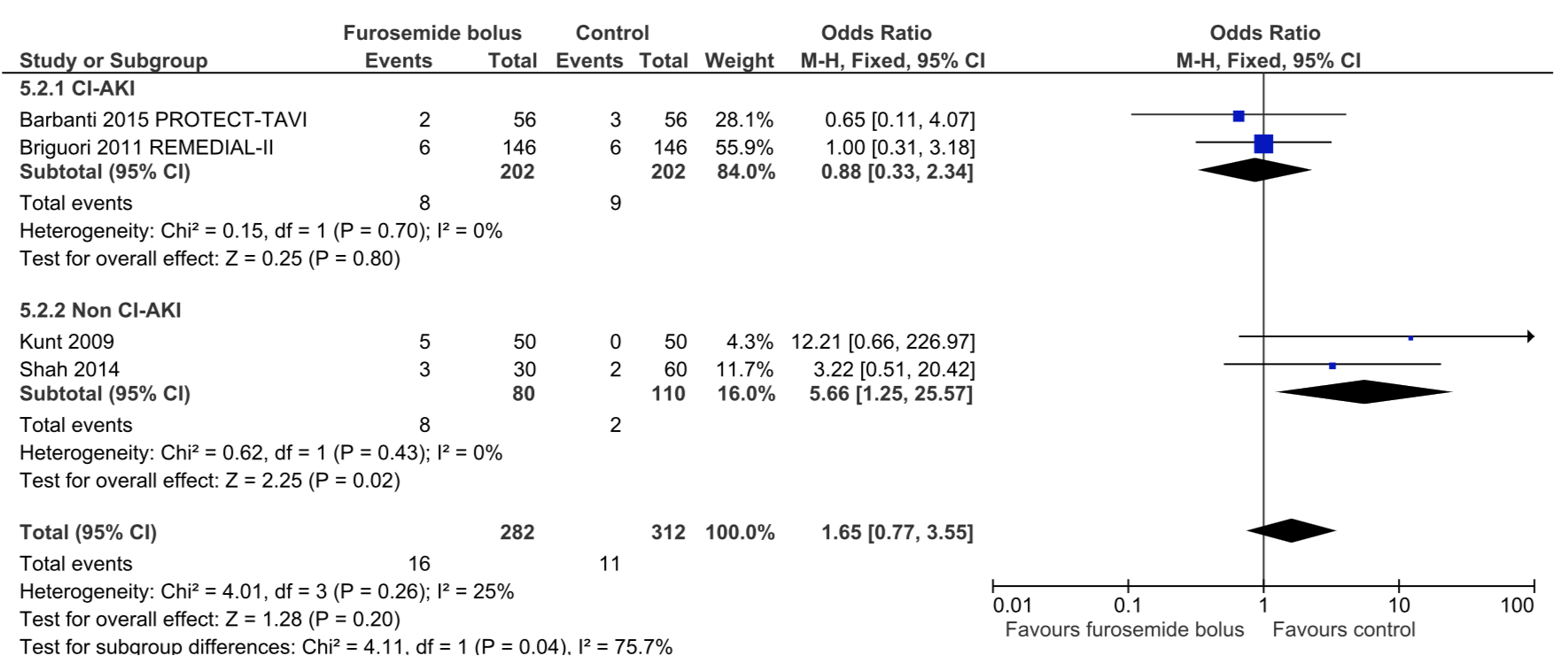
**

**Supplementary Figure I.** Forest plot for 28/30-days mortality at the longest follow-up available, stratified by treatment indication

**Supplementary Figure J.** Forest plot for AKI

**Supplementary Figure K.** Forest plot for AKI, stratified by control treatment

**Supplementary Figure L.** Forest plot for AKI, stratified by setting

**
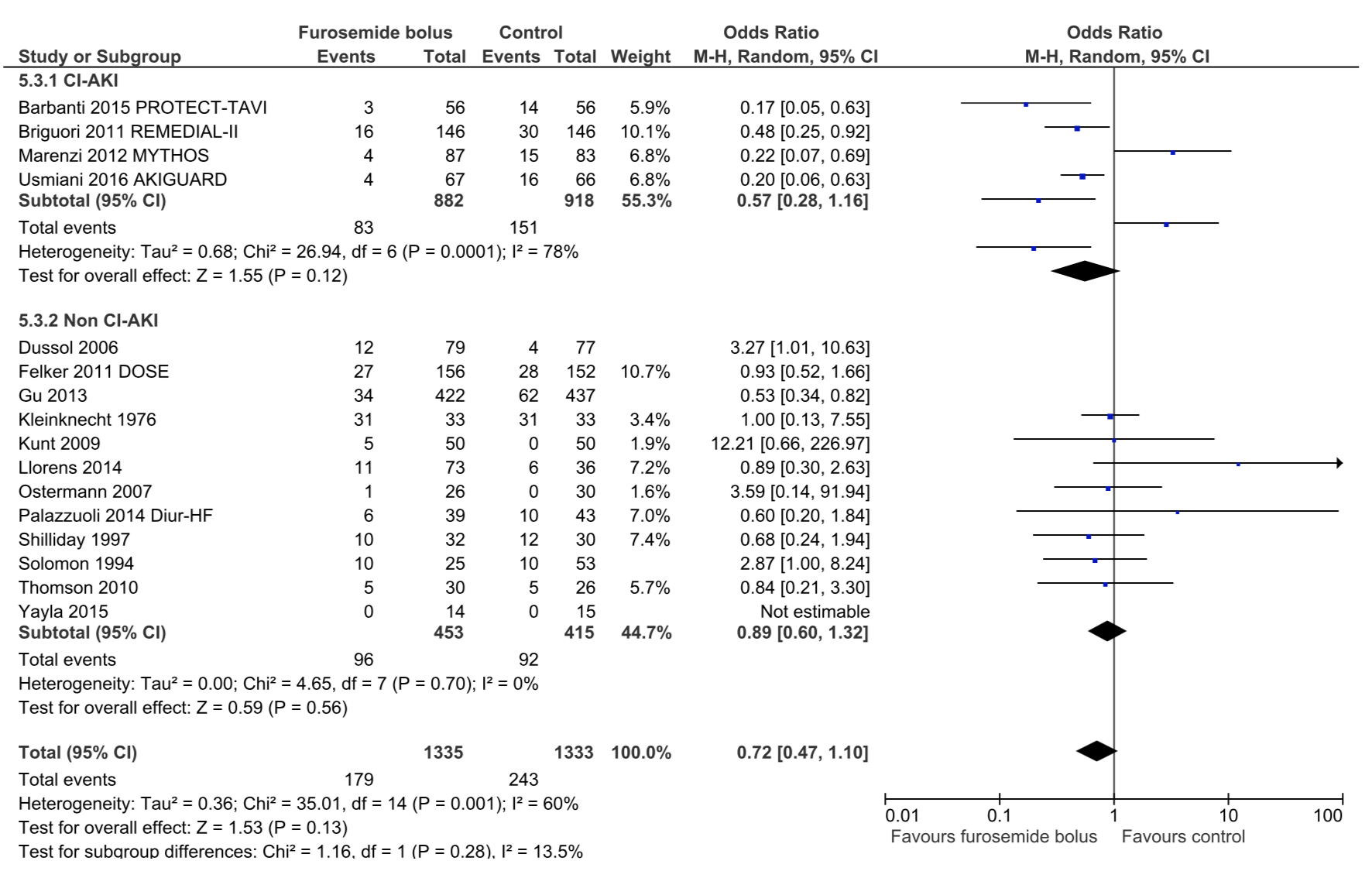
**

**Supplementary Figure M.** Forest plot for AKI, stratified by treatment indication

**Supplementary Figure N.** Forest plot for receipt of renal-replacement therapy

**Supplementary Figure O.** Forest plot for receipt of renal-replacement therapy, stratified by control treatment

**Supplementary Figure P.** Forest plot for receipt of renal-replacement therapy, stratified by setting

**
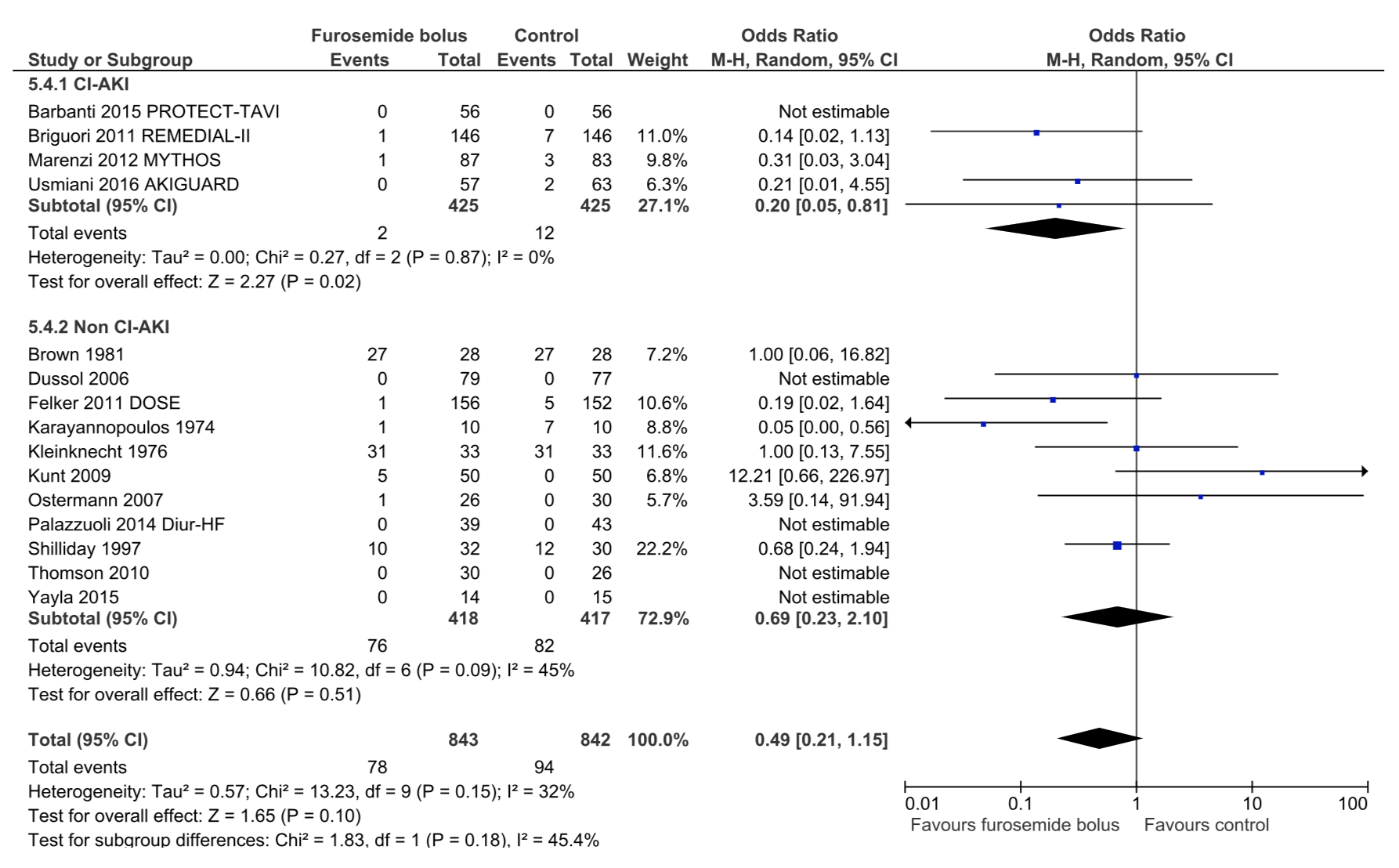
**

**Supplementary Figure Q.** Forest plot for receipt of renal-replacement therapy, stratified by treatment indication

**Supplementary Figure R.** Forest plot for length of hospital stay

**Supplementary Figure S.** Forest plot for length of hospital stay, stratified by control treatment

**Supplementary Figure T.** Forest plot for length of hospital stay, stratified by setting

**
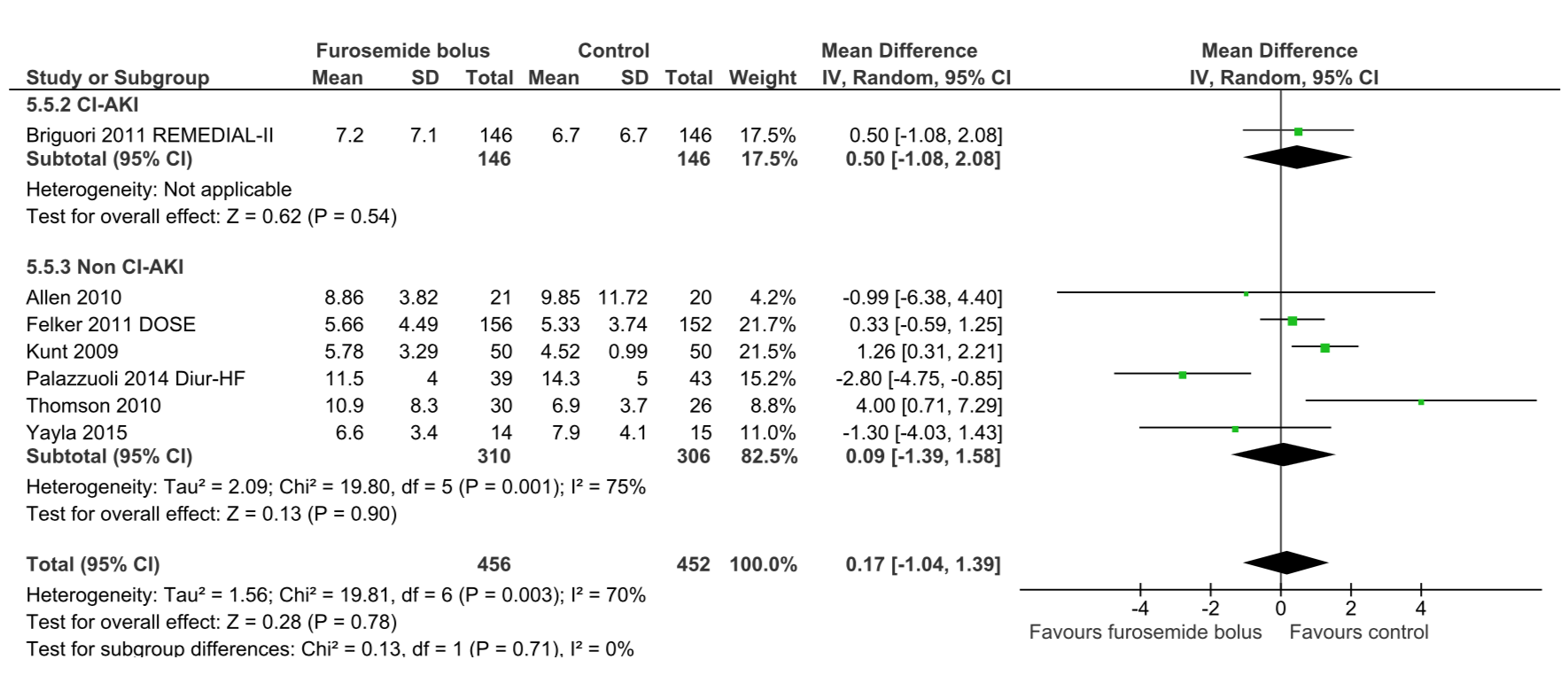
**

**Supplementary Figure U.** Forest plot for length of hospital stay, stratified by treatment indication

**Supplementary Figure V.** Forest plot for peak serum creatinine after randomization

**Supplementary Figure W.** Forest plot for peak serum creatinine after randomization, stratified by control treatment

**Supplementary Figure X.** Forest plot for peak serum creatinine after randomization, stratified by setting

**Supplementary Figure Y.** Forest plot for peak serum creatinine after randomization, stratified by treatment indication

**Supplementary Figure Z.** Forest plot for all-cause mortality, contrast-induced AKI versus non contrast-induced AKI.


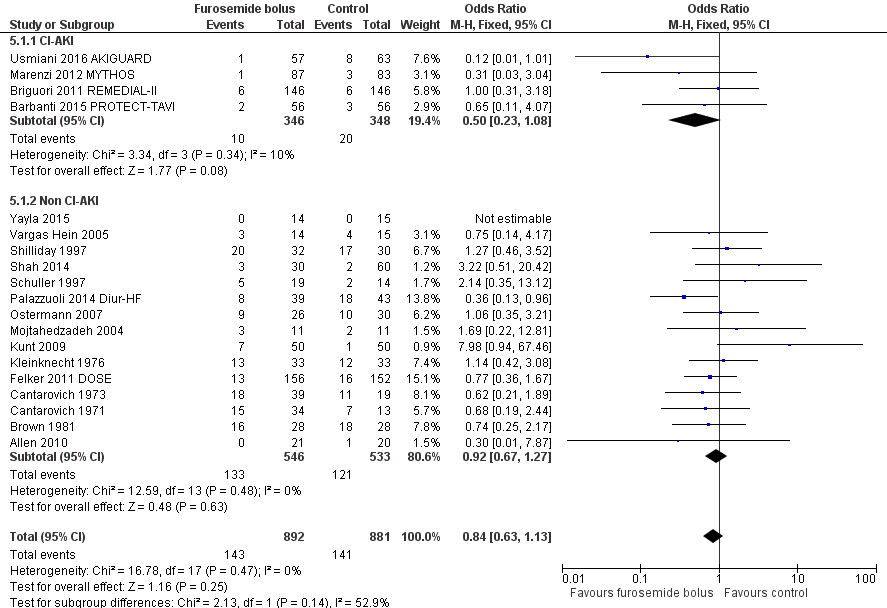


**Supplementary Figure AB.** Forest plot for all-cause 28/30-days mortality, contrast-induced AKI versus non contrast-induced AKI.

**
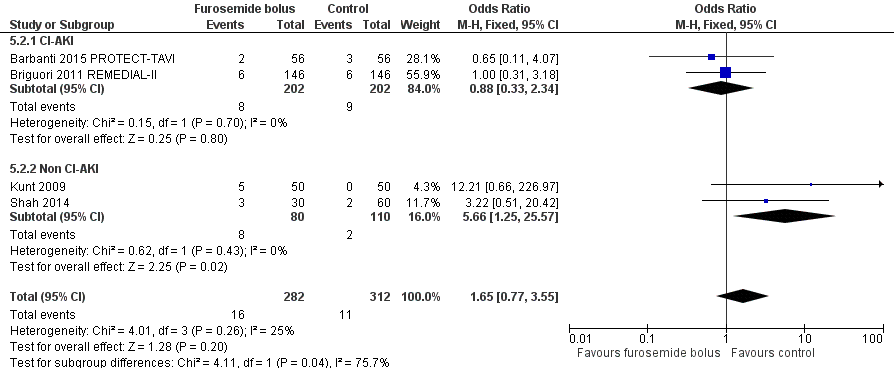
**

**Supplementary Figure AC.** Forest plot for AKI, contrast-induced AKI versus non contrast-induced AKI

**
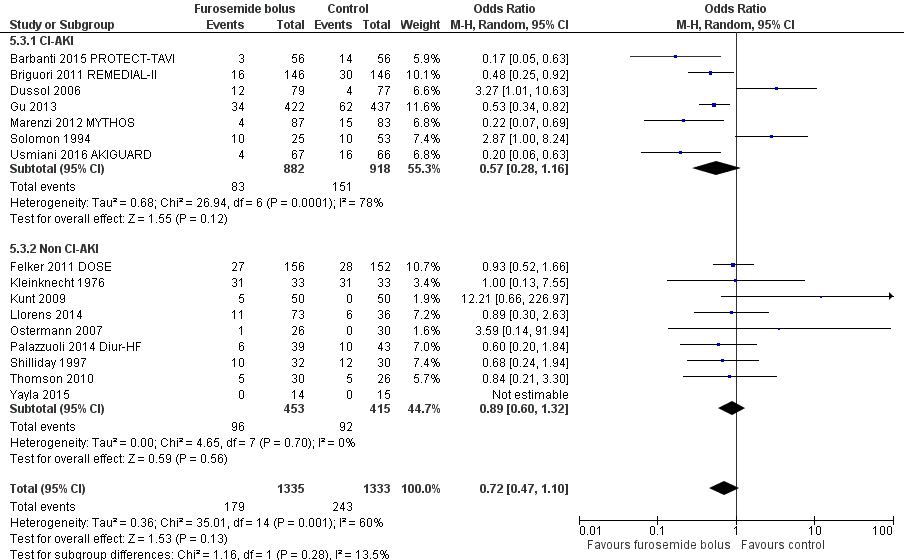
**

**Supplementary Figure AD.** Forest plot for receipt of renal-replacement therapy, contrast-induced AKI versus non contrast-induced AKI


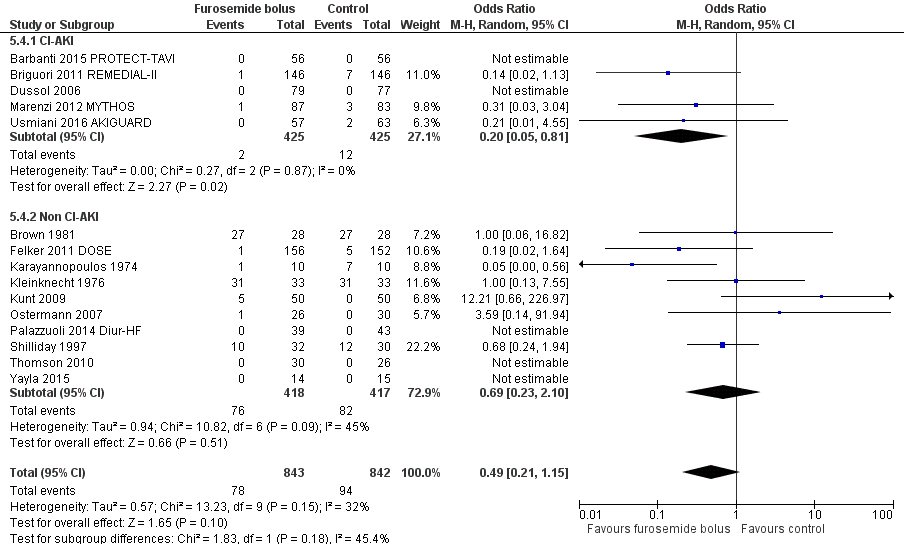


**Supplementary Figure AE.** Forest plot for hospital length of stay, contrast-induced AKI versus non contrast-induced AKI


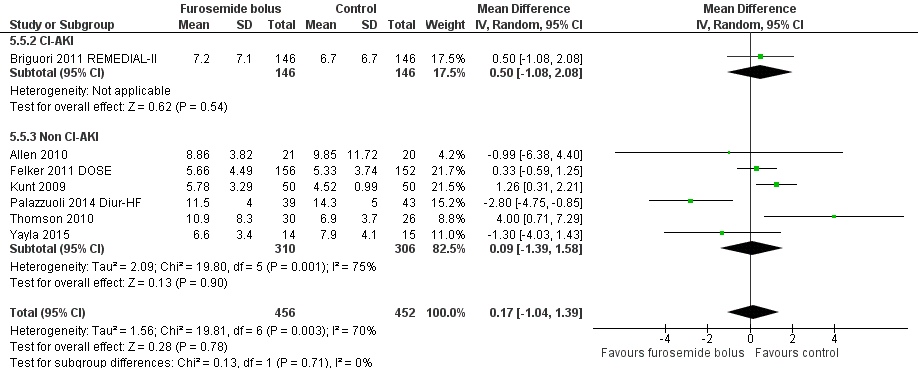


**Supplementary Figure AF.** Forest plot for peak serum creatinine, contrast-induced AKI versus non contrast-induced AKI

**
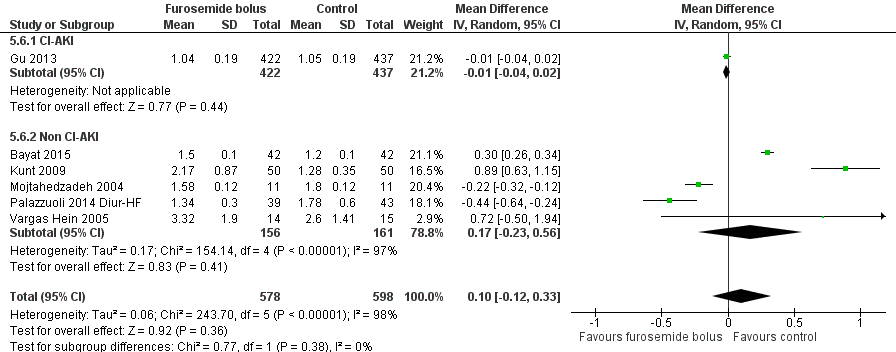
**

**Supplementary References**

1. Badawy SS, Fahmy A. Efficacy and cardiovascular tolerability of continuous veno-venous hemodiafiltration in acute decompensated heart failure: a randomized comparative study. J Crit Care. 2012 Feb;27(1):106.
2. Bagshaw SM, Gibney RTN, Kruger P, Hassan I, McAlister FA, Bellomo R. The effect of low-dose furosemide in critically ill patients with early acute kidney injury: A pilot randomized blinded controlled trial (the SPARK study). J Crit Care. 2017 Jul 12;42:138-146.
3. Bagshaw SM, Gibney RT, McAlister FA, Bellomo R. The SPARK Study: a phase II randomized blinded controlled trial of the effect of furosemide in critically ill patients with early acute kidney injury. Trials. 2010 May 11;11:50.
4. Bart BA, Boyle A, Bank AJ, Anand I, Olivari MT, Kraemer M et al. Ultrafiltration versus usual care for hospitalized patients with heart failure: the Relief for Acutely Fluid-Overloaded Patients With Decompensated Congestive Heart Failure (RAPID-CHF) trial. J Am Coll Cardiol. 2005 Dec 6;46(11):2043-6.
5. Bart BA, Goldsmith SR, Lee KL, Givertz MM, O'Connor CM, Bull DA et al; Heart Failure Clinical Research Network. Ultrafiltration in decompensated heart failure with cardiorenal syndrome. N Engl J Med. 2012 Dec 13;367(24):2296-304.
6. Cantarovich F, Rangoonwala B, Lorenz H, Verho M, Esnault VL; High-Dose Flurosemide in Acute Renal Failure Study Group. High-dose furosemide for established ARF: a prospective, randomized, double-blind, placebo-controlled, multicenter trial. Am J Kidney Dis. 2004 Sep;44(3):402-9.
7. Costanzo MR, Guglin ME, Saltzberg MT, Jessup ML, Bart BA, Teerlink JR et al; UNLOAD Trial Investigators. Ultrafiltration versus intravenous diuretics for patients hospitalized for acute decompensated heart failure. J Am Coll Cardiol. 2007 Feb 13;49(6):675-83.
8. Costanzo MR, Saltzberg MT, Jessup M, Teerlink JR, Sobotka PA; Ultrafiltration Versus Intravenous Diuretics for Patients Hospitalized for Acute Decompensated Heart Failure (UNLOAD) Investigators. Ultrafiltration is associated with fewer rehospitalizations than continuous diuretic infusion in patients with decompensated heart failure: results from UNLOAD. J Card Fail. 2010 Apr;16(4):277-84.
9. Cotter G, Weissgarten J, Metzkor E, Moshkovitz Y, Litinski I, Tavori Uet al. Increased toxicity of high-dose furosemide versus low-dose dopamine in the treatment of refractory congestive heart failure. Clin Pharmacol Ther. 1997 Aug;62(2):187-93.
10. Fakhari S, Bavil FM, Bilehjani E, Abolhasani S, Mirinazhad M, Naghipour B. Prophylactic furosemide infusion decreasing early major postoperative renal dysfunction in on-pump adult cardiac surgery: a randomized clinical trial. Res Rep Urol. 2017 Jan 19;9:5-13.
11. Giglioli C, Landi D, Cecchi E, Chiostri M, Gensini GF, Valente S et al. Effects of ULTRAfiltration vs. DIureticS on clinical, biohumoral and haemodynamic variables in patients with deCOmpensated heart failure: the ULTRADISCO study. Eur J Heart Fail. 2011 Mar;13(3):337-46.
12. Giamouzis G, Butler J, Starling RC, Karayannis G, Nastas J, Parisis C et al. Impact of dopamine infusion on renal function in hospitalized heart failure patients: results of the Dopamine in Acute Decompensated Heart Failure (DAD-HF) Trial. J Card Fail. 2010 Dec;16(12):922-30.
13. Hager B, Betschart M, Krapf R. Effect of postoperative intravenous loop diuretic on renal function after major surgery. Schweiz Med Wochenschr. 1996 Apr 20;126(16):666-73.
14. Hamishehkar H, Sanaie S, Fattahi V, Mesgari M, Mahmoodpoor A. The Effect of Furosemide on the Level of Neutrophil Gelatinase-associated Lipocalin in Critically Hospitalized Patients with Acute Kidney Injury. Indian J Crit Care Med. 2017 Jul;21(7):442-447.
15. Hanna MA, Tang WH, Teo BW, O'Neill JO, Weinstein DM, Lau SM et al. Extracorporeal ultrafiltration vs. conventional diuretic therapy in advanced decompensated heart failure. Congest Heart Fail. 2012 Jan-Feb;18(1):54-63.
16. Khan NA, Campbell NR, Frost SD, Gilbert K, Michota FA, Usmani A et al. Risk of intraoperative hypotension with loop diuretics: a randomized controlled trial. Am J Med. 2010 Nov;123(11):1059.e1-8.
17. Klinge JM, Scharf J, Hofbeck M, Gerling S, Bonakdar S, Singer H. Intermittent administration of furosemide versus continuous infusion in the postoperative management of children following open heart surgery. Intensive Care Med. 1997 Jun;23(6):693-7.
18. Kwiatkowski DM, Krawczeski CD. Acute kidney injury and fluid overload in infants and children after cardiac surgery. Pediatr Nephrol. 2017 Mar 30.
19. Lassnigg A, Donner E, Grubhofer G, Presterl E, Druml W, Hiesmayr M. Lack of renoprotective effects of dopamine and furosemide during cardiac surgery. J Am Soc Nephrol. 2000 Jan;11(1):97-104.
20. Licata G, Di Pasquale P, Parrinello G, Cardinale A, Scandurra A, Follone G et al. Effects of high-dose furosemide and small-volume hypertonic saline solution infusion in comparison with a high dose of furosemide as bolus in refractory congestive heart failure: long-term effects. Am Heart J. 2003 Mar;145(3):459-66.
21. Lim E, Ali ZA, Attaran R, Cooper G. Evaluating routine diuretics after coronary surgery: a prospective randomized controlled trial. Ann Thorac Surg. 2002 Jan;73(1):153-5.
22. Luciani GB, Nichani S, Chang AC, Wells WJ, Newth CJ, Starnes VA. Continuous versus intermittent furosemide infusion in critically ill infants after open heart operations. Ann Thorac Surg. 1997 Oct;64(4):1133-9.
23. Majumdar SR, Kjellstrand CM, Tymchak WJ, Hervas-Malo M, Taylor DA, Teo KK. Forced euvolemic diuresis with mannitol and furosemide for prevention of contrast-induced nephropathy in patients with CKD undergoing coronary angiography: a randomized controlled trial. Am J Kidney Dis. 2009 Oct;54(4):602-9.
24. Malfatto G, Della Rosa F, Villani A, Rella V, Branzi G, Facchini M, Parati G. Intermittent levosimendan infusions in advanced heart failure: favourable effects on left ventricular function, neurohormonal balance, and one-year survival. J Cardiovasc Pharmacol. 2012 Nov;60(5):450-5.
25. Nuutinen L, Hollmén A. The effect of prophylactic use of furosemide on renal function during open heart surgery. Ann Chir Gynaecol. 1976;65(4):258-66.
26. Nuutinen LS, Kairaluoma M, Tuononen S, Larmi TK. The effect of furosemide on renal function in open heart surgery. J Cardiovasc Surg (Torino). 1978 Sep-Oct;19(5):471-9.
27. Palazzuoli A, Pellegrini M, Franci B, Beltrami M, Ruocco G, Gonnelli S et al. Short and long-term effects of continuous versus intermittent loop diuretics treatment in acute heart failure with renal dysfunction. Intern Emerg Med. 2015 Feb;10(1):41-9.
28. Paterna S, Di Pasquale P, Parrinello G, Amato P, Cardinale A et al. Effects of high-dose furosemide and small-volume hypertonic saline solution infusion in comparison with a high dose of furosemide as a bolus, in refractory congestive heart failure. Eur J Heart Fail. 2000 Sep;2(3):305-13.
29. Rogers HL, Marshall J, Bock J, Dowling TC, Feller E, Robinson S, Gottlieb SS. A randomized, controlled trial of the renal effects of ultrafiltration as compared to furosemide in patients with acute decompensated heart failure. J Card Fail. 2008 Feb;14(1):1-5.
30. Shemirani H, Pourrmoghaddas M. A randomized trial of saline hydration to prevent contrast-induced nephropathy in patients on regular captopril or furosemide therapy undergoing percutaneous coronary intervention. Saudi J Kidney Dis Transpl. 2012 Mar;23(2):280-5.
31. Singh NC, Kissoon N, al Mofada S, Bennett M, Bohn DJ. Comparison of continuous versus intermittent furosemide administration in postoperative pediatric cardiac patients. Crit Care Med. 1992 Jan;20(1):17-21.
32. Sirivella S, Gielchinsky I, Parsonnet V. Mannitol, furosemide, and dopamine infusion in postoperative renal failure complicating cardiac surgery. Ann Thorac Surg. 2000 Feb;69(2):501-6.
33. Triposkiadis FK, Butler J, Karayannis G, Starling RC, Filippatos G, Wolski K et al. Efficacy and safety of high dose versus low dose furosemide with or without dopamine infusion: the Dopamine in Acute Decompensated Heart Failure II (DAD-HF II) trial. Int J Cardiol. 2014 Mar 1;172(1):115-21.
34. van der Voort PH, Boerma EC, Koopmans M, Zandberg M, de Ruiter J, Gerritsen RT et al. Furosemide does not improve renal recovery after hemofiltration for acute renal failure in critically ill patients: a double blind randomized controlled trial. Crit Care Med. 2009 Feb;37(2):533-8.
35. Weisberg LS, Kurnik PB, Kurnik BR. Risk of radiocontrast nephropathy in patients with and without diabetes mellitus. Kidney Int. 1994 Jan;45(1):259-65.
36. Allen LA, Turer AT, Dewald T, Stough WG, Cotter G, O'Connor CM. Continuous versus bolus dosing of Furosemide for patients hospitalized for heart failure. Am J Cardiol. 2010;105:1794-7.
37. Barbanti M, Gulino S, Capranzano P, Immè S, Sgroi C, Tamburino C, et al. Acute Kidney Injury With the RenalGuard System in Patients Undergoing Transcatheter Aortic Valve Replacement: The PROTECT-TAVI Trial (PROphylactic effecT of furosEmide-induCed diuresis with matched isotonic intravenous hydraTion in Transcatheter Aortic Valve Implantation). JACC Cardiovasc Interv. 2015;8:1595-604.
38. Briguori C, Visconti G, Focaccio A, Airoldi F, Valgimigli M, Sangiorgi GM, et al; REMEDIAL II Investigators. Renal Insufficiency After Contrast Media Administration Trial II (REMEDIAL II): RenalGuard System in high-risk patients for contrast-induced acute kidney injury. Circulation. 2011;124:1260-9.
39. Brown CB, Ogg CS, Cameron JS. High dose frusemide in acute renal failure: a controlled trial. Clin Nephrol. 1981;15:90-6.
40. Cantarovich F, Fernandez JC, Locatelli A, Perez Loredo J. Frusemide in high doses in the treatment of acute renal failure. Postgrad Med J. 1971;47:Suppl:13-7.
41. Cantarovich F, Galli C, Benedetti L, Chena C, Castro L, Correa C, et al. High dose frusemide in established acute renal failure. Br Med J. 1973;4:449-50.
42. Felker GM, Lee KL, Bull DA, Redfield MM, Stevenson LW, Goldsmith SR, et al; NHLBI Heart Failure Clinical Research Network. Diuretic strategies in patients with acute decompensated heart failure. N Engl J Med. 2011;364:797-805.
43. Kleinknecht D, Ganeval D, Gonzalez-Duque LA, Fermanian J. Furosemide in acute oliguric renal failure. A controlled trial. Nephron. 1976;17:51-8.
44. Kunt AT, Akgün S, Atalan N, Bitir N, Arsan S. Furosemide infusion prevents the requirement of renal replacement therapy after cardiac surgery. Anadolu Kardiyol Derg. 2009;9:499-504.
45. Marenzi G, Ferrari C, Marana I, Assanelli E, De Metrio M, Teruzzi G,. Prevention of contrast nephropathy by furosemide with matched hydration: the MYTHOS (Induced Diuresis With Matched Hydration Compared to Standard Hydration for Contrast Induced Nephropathy Prevention) trial. JACC Cardiovasc Interv. 2012;5:90-7.
46. Mojtahedzadeh M, Salehifar E, Vazin A, Mahidiani H, Najafi A, Tavakoli M, et al. Comparison of hemodynamic and biochemical effects of furosemide by continuous infusion and intermittent bolus in critically ill patients. J Infus Nurs. 2004;27:255-61.
47. Ostermann M, Alvarez G, Sharpe MD, Martin CM. Frusemide administration in critically ill patients by continuous compared to bolus therapy. Nephron Clin Pract. 2007;107:c70-6.
48. Palazzuoli A, Pellegrini M, Ruocco G, Martini G, Franci B, Campagna MS, et al. Continuous versus bolus intermittent loop diuretic infusion in acutely decompensated heart failure: a prospective randomized trial. Crit Care. 2014;18:R134.
49. Schuller D, Lynch JP, Fine D. Protocol-guided diuretic management: comparison of furosemide by continuous infusion and intermittent bolus. Crit Care Med. 1997;25:1969-75.
50. Shah RA, Subban V, Lakshmanan A, Narayanan S, Udhayakumaran K, Pakshirajan B, et al. A prospective, randomized study to evaluate the efficacy of various diuretic strategies in acute decompensated heart failure. Indian Heart J. 2014;66:309-16.
51. Shilliday IR, Quinn KJ, Allison ME. Loop diuretics in the management of acute renal failure: a prospective, double-blind, placebo-controlled, randomized study. Nephrol Dial Transplant. 1997;12:2592-6.
52. Usmiani T, Andreis A, Budano C, Sbarra P, Andriani M, Garrone P, et al. AKIGUARD (Acute Kidney Injury GUARding Device) trial: in-hospital and one-year outcomes. J Cardiovasc Med (Hagerstown). 2016;17:530-7.
53. Vargas Hein O, Staegemann M, Wagner D, von Heymann C, Martin M, Morgera S, et al. Torsemide versus furosemide after continuous renal replacement therapy due to acute renal failure in cardiac surgery patients. Ren Fail. 2005;27:385-92.
54. Yayla Ç, Akyel A, Canpolat U, Gayretli Yayla K, Eyiol A, Akboğa MK, et al. Comparison of three diuretic treatment strategies for patients with acute decompensated heart failure. Herz. 2015;40:1115-20.
55. Llorens P, Miró Ò, Herrero P, Martín-Sánchez FJ, Jacob J, Valero Aet al. Clinical effects and safety of different strategies for administering intravenous diuretics in acutely decompensated heart failure: a randomised clinical trial. Emerg Med J. 2014;31:706-13.
56. Thomson MR, Nappi JM, Dunn SP, Hollis IB, Rodgers JE, Van Bakel AB. Continuous versus intermittent infusion of furosemide in acute decompensated heart failure. J Card Fail. 2010;16:188-93.
57. Dussol B, Morange S, Loundoun A, Auquier P, Berland Y. A randomized trial of saline hydration to prevent contrast nephropathy in chronic renal failure patients. Nephrol Dial Transplant. 2006;21:2120-6.
58. Gu GQ, Lu R, Cui W, Liu F, Zhang Y, Yang XH, et al. Low-dose furosemide administered with adequate hydration reduces contrast-induced nephropathy in patients undergoing coronary angiography. Cardiology. 2013;125:69-73.
59. Solomon R, Werner C, Mann D, D'Elia J, Silva P. Effects of saline, mannitol, and furosemide on acute decreases in renal function induced by radiocontrast agents. N Engl J Med. 1994;331:1416-20.
60. Karayannopoulos S. Letter: High-dose frusemide in renal failure. Br Med J. 1974; 2: 278-279.
